# Antiviral Profiling of C-18- or C-19-Functionalized Semisynthetic Abietane Diterpenoids

**DOI:** 10.1021/acs.jnatprod.2c00464

**Published:** 2022-08-15

**Authors:** Miguel A. González-Cardenete, Damir Hamulić, Francisco J. Miquel-Leal, Natalia González-Zapata, Orlando J. Jimenez-Jarava, Yaneth M. Brand, Laura C. Restrepo-Mendez, Marlen Martinez-Gutierrez, Liliana A. Betancur-Galvis, Maria L. Marín

**Affiliations:** † Instituto de Tecnología Química (UPV-CSIC), Universitat Politècnica de Valencia-Consejo Superior de Investigaciones Científicas, Avda de los Naranjos s/n, 46022 Valencia, Spain; § Grupo de Investigaciones Dermatológicas, Instituto de Investigaciones Médicas, Facultad de Medicina, Universidad de Antioquia, 050010 Medellín, Colombia; ⊥ Grupo de Investigación en Ciencias Animales-GRICA, Universidad Cooperativa de Colombia, 680001 Bucaramanga, Colombia; ‡ Línea de Descubrimiento y Evaluación de Compuestos Antivirales, Grupo de Investigación en Microbiología Básica y Aplicada (MICROBA), Escuela de Microbiología, Universidad de Antioquia, 050010 Medellín, Colombia

## Abstract

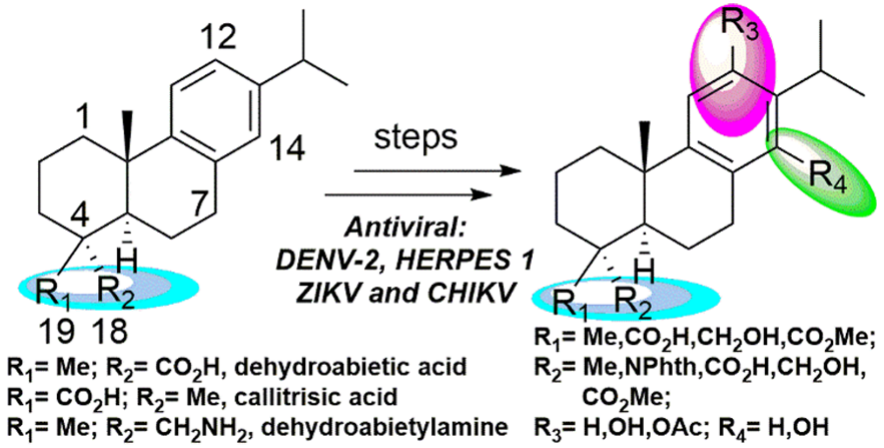

Viral infections affect several million patients annually. Although hundreds of viruses are known to be pathogenic, only a few can be treated in the clinic with available antiviral drugs. Naturally based pharmacotherapy may be a proper alternative for treating viral diseases. Several natural and semisynthetic abietane-type diterpenoids have shown important antiviral activities. In this study, a biological evaluation of a number of either C-18- or C-19-functionalized known semisynthetic abietanes against *Zika virus*, *Dengue virus*, *Herpes virus simplextype* 1, and *Chikungunya virus* are reported. Semisynthetic abietane ferruginol and its analogue 18-(phthalimid-2-yl)ferruginol displayed broad-spectrum antiviral properties. The scale-up synthesis of this analogue has been optimized for further studies and development. This molecule displayed an EC_50_ between 5.0 and 10.0 μM against Colombian Zika virus strains and EC_50_ = 9.8 μM against Chikungunya virus. Knowing that this ferruginol analogue is also active against *Dengue virus type* 2 (EC_50_ = 1.4 μM, DENV-2), we can conclude that this compound is a promising broad-spectrum antiviral agent paving the way for the development of novel antivirals.

Viral infections affect several million patients annually, and only a few can be treated in the clinic with available antiviral drugs. However, for some very pathogenic viruses such as Zika (ZIKV), Ebola (EBOV), severe acute respiratory syndrome (SARS), and others, there are still no drugs on the market against them.^1^ While commonly used antivirals often show limited efficacy and serious adverse effects, herbal extracts have been in use for medicinal purposes since ancient times and are known for their antiviral properties and more tolerable side effects. Thus, naturally based pharmacotherapy may be a proper alternative for treating viral diseases.^2^ Chemical diversity in natural products widely attracts scientific attention to find potential therapeutic agents like anticancer and anti-infective drugs from natural sources. Around 50 percent of commercial anti-infective drugs are either natural products (NPs), directly derived from NPs, or inspired by NP structures, of which one-third are antiviral agents.^3^

Dengue is the most common mosquito-borne viral disease in the Americas and the most suspected in patients with fever. However, the recent introduction of two new arboviral diseases [chikungunya virus (CHIKV) in late 2013 and ZIKV in 2014] has created a new challenge for public health in the Americas.^4^ The three arboviral diseases (dengue, chikungunya, and Zika) can produce very similar clinical symptoms, mainly during the acute phase (the first days of the disease), hindering clinical diagnosis by health workers, creating problems for appropriate case management, and sometimes triggering fatal events. Diseases caused by arboviruses usually constitute a syndrome that can be either febrile (e.g., dengue and chikungunya) or exanthematic (Zika).^4^ Other frequent symptoms are headache and body pain, including myalgia and manifestations in the joints.

While the majority of cases of arboviral disease are self-limiting, sometimes they can manifest severe forms, such as shock, hemorrhage, or severe organ damage (in the case of dengue) or neurological complications (Zika), which can lead to death.^4^ CHIKV infection can also be clinically severe, particularly at the extreme ages of life. Chikungunya patients can develop postacute or chronic arthropathy lasting 21 to 90 days in acute cases and 3 months to ≥2 years in chronic cases.^4^ Furthermore, these three arboviral diseases can cause autoimmune disease affecting the central nervous system (CNS). For example, Guillain-Barré syndrome (GBS) or encephalopathy and visual damage due to optic neuritis.^4^

Abietane diterpenoids are naturally occurring metabolites isolated from a large variety of terrestrial plants that show a wide range of promising biological activities, including antiviral properties.^5^ Several research groups have explored the potential as chemotherapeutic agents of abietanes by means of derivatives by semisynthesis from commercial starting materials such as (−)-abietic acid (**1**), transformable into (+)-dehydroabietic acid (**2**), and (+)-dehydroabietylamine (**3**, DHAA), also called leelamine.^6^ These materials are produced in the industry of pine oleoresin, which has a world production of more than one million metric tons per year. To date, there is only one commercial drug based on abietane-type diterpenoids, ecabet sodium (**4**), commercialized as Ecabet. Nevertheless, there are ongoing clinical trials with related molecules such as tanshinones, first isolated from the roots of *Salvia miltiorrhiza* “tanshen”, a well-known Traditional Chinese Medicine.

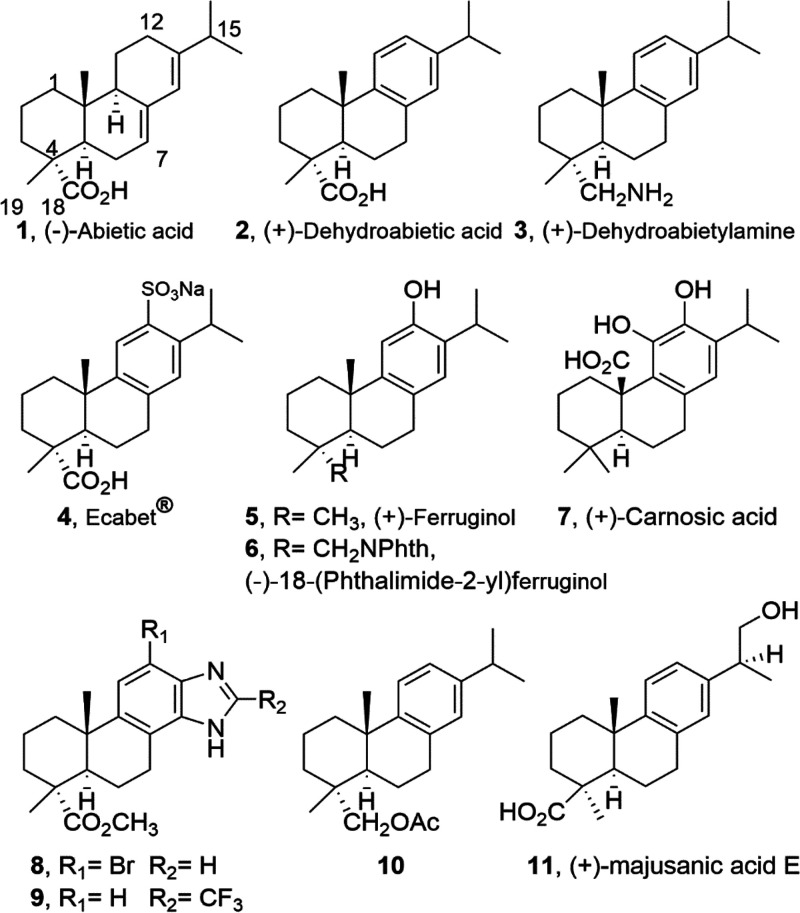



As an example of antiviral abietanes, (+)-ferruginol (**5**) has displayed anti-SARS with strong cytopathogenic effect (CPE) reduction and potent replication inhibition, as have other abietane congeners.^7^ Its semisynthetic phthalimide analogue **6** has exhibited anti-dengue and anti-herpes properties.^8^ Compound **6** has led to two patents as an antiviral agent,^9,10^ and some of its synthetic intermediates have shown potent antimalarial properties.^11^ Natural (+)-carnosic acid (**7**) has shown inhibitory effects on HIV-1 protease and HIV-1 virus^12^ and human respiratory syncytial virus replication.^13^ Semisynthetic benzimidazoles **8** and **9** inhibited both varicella-zoster virus (VZV) and cytomegalovirus (CMV) replication.^14^ Semisynthetic dehydroabietinol acetate (**10**) exhibited mild anti-herpes activity.^15^ In recent years, a number of C-19-functionalized abietane acids (i.e., majusanic acid E, **11**, and its congeners (angustanoic and jiadifenoic acids)) have been isolated from Illiciaceae plants showing important anti-Coxsackie virus activities.^16^ Other related abietane congeners possessing an α,β-unsaturated γ-lactone moiety isolated from *Euphorbia neriifolia* have exhibited significant anti-HIV properties.^17^

A recent computational study using a molecular docking approach predicts that several abietane diterpenoids are good selective inhibitors of a key protease, such as alphavirus nonstructural protein 2 (nsP2), which is a promising target in antiviral drug development.^18^ Also, very recently it has been reported that drugs inhibiting the intracellular cholesterol transport possess broad-spectrum antiviral activity for CHIKV and several Flaviviridae family members, including Zika, West Nile, and dengue virus.^19^ Leelamine (**3**), a typical aromatic abietane, has demonstrated cancer cell death by inhibiting intracellular cholesterol transport,^20^ for which we foresee potential broad-spectrum antiviral properties in other abietane analogues.

Having in mind this background of antiviral activities of abietane-based diterpenoids, in this work, we have expanded the knowledge of antiviral properties of several available C-18- and C-19-functionalized abietane diterpenoids. After our development, in 2012, of the synthesis of (+)-ferruginol (**5**) starting from the commercially available (+)-dehydroabietylamine (**3**), ferruginol analogue **6** was prepared.^21^ It was envisaged that several readily available ferruginol analogues could have antiviral properties, and thus, compound **6** was discovered in 2016 as an anti-dengue and anti-herpetic agent.^8^ Thus, in continuation of our work on bioactive diterpenoids, we report additional data on the antiviral activities of ferruginol (**5**) and its corresponding C-18 phthalimide analogue **6**, three semisynthetic dehydroabietic acid derivatives (**12**–**14**, C-18-substitution), and several ferruginol analogues (**15**–**26**) (Scheme 1) against *Zika virus*, *Dengue virus*, *Herpes virus simplex type* 1, and *Chikungunya virus*.

**Scheme 1:**
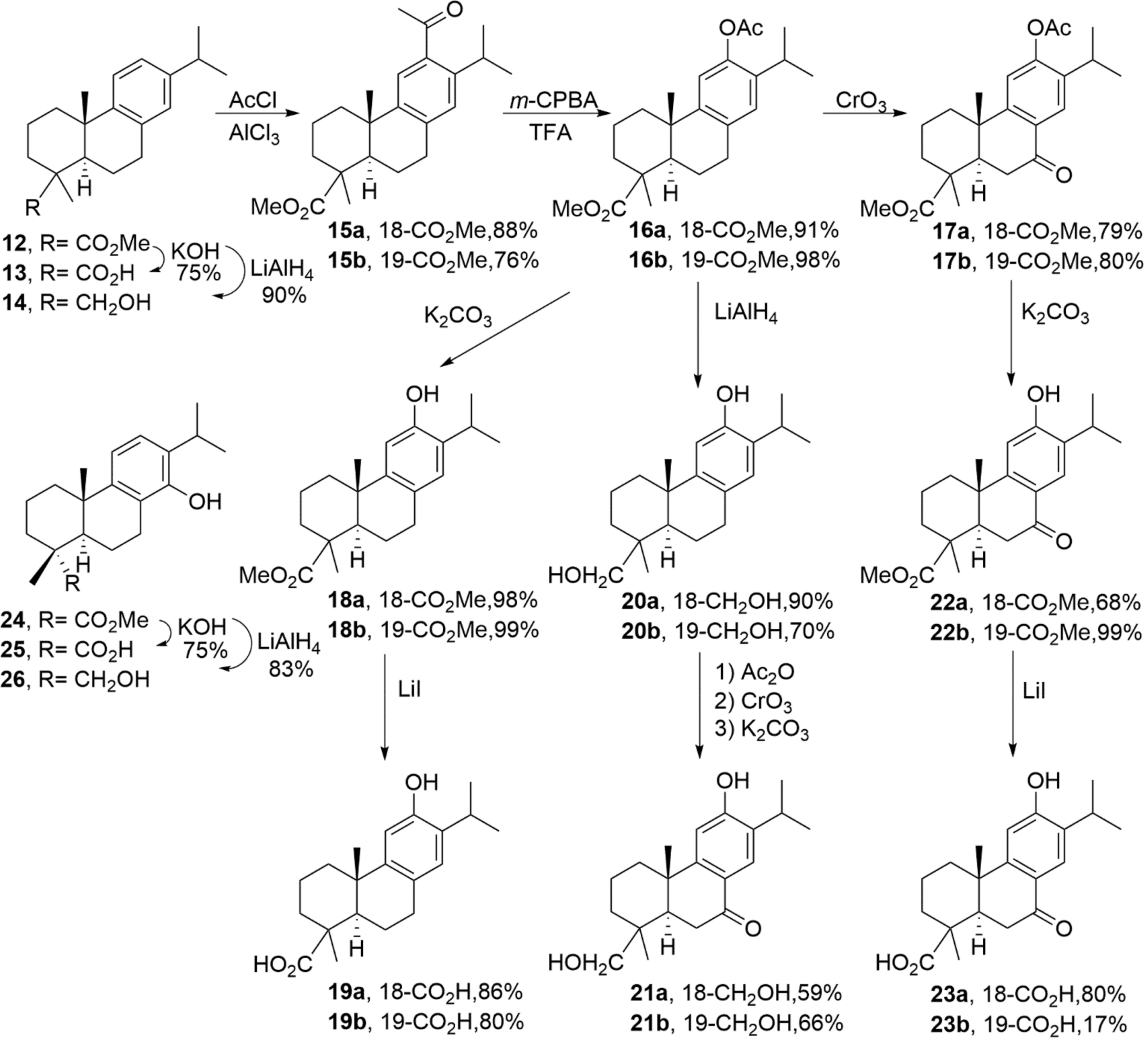
Synthetic Route for the Preparation of Tested Molecules **12**–**26**

For the synthesis of compound **6**, we optimized the synthesis procedure reported by Waldvogel and co-workers for compound **6** from ca. 65% (+)-dehydroabietylamine (**3**) in four synthetic steps in multigram scale.^22^ In this work, we propose an optimized experimental procedure to obtain in multigram scale this broad-spectrum antiviral and improved synthesis of (+)-ferruginol (**5**) in small scale. The antiviral investigation has been extended, and additional data are herein reported along with some structure–activity relationships for the different viruses.

## Results and Discussion

### Chemistry

Based on our previous work, the tested compounds **12**–**26** were obtained following synthetic routes reported by us.^15,23−25^ Compounds **12**–**14**^15^ and **24**–**26**^23^ were obtained from commercially available (−)-abietic acid as well as compounds **15a**–**23a**,^24^ whose intermediate was methyl dehydroabietate (**12**, R = 18-CO_2_Me) (Scheme 1) obtained from (−)-abietic acid by esterification and aromatization with a Pd/C catalyst.^15^ Compounds **15b**–**23b**^25^ were obtained in a similar manner, but in this case starting from (+)-methyl callitrisate (**12**, R = 19-CO_2_Me) (Scheme 1) extracted from Sandarac resin.^25^ To generate the C-12 phenolic moiety, Friedel–Crafts and Baeyer–Villiger reactions were used (Scheme 1). Functional group manipulation was carried out under standard conditions except for the hydrolysis of selected ester groups, which was executed by nucleophilic cleavage with LiI. Several of the obtained compounds are naturally occurring such as 12-hydroxydehydroabietic acid (**19a**), lambertic acid (**19b**), 18-hydroxyferruginol (**20a**), 19-hydroxyferruginol (**20b**), and liquiditerpenoic acid A (**23a**), and some may be prepared by other methods. (+)-Ferruginol (**5**) was prepared in this work in a slightly better yield from 12-hydroxydehydroabietylamine^21^ by deamination with more equivalents of hydroxylamine sulfonic acid over a longer reaction time. For a 100 mg scale we obtained ca. 60% yield instead of the original 40% in multigram scale.

**Scheme 2:**
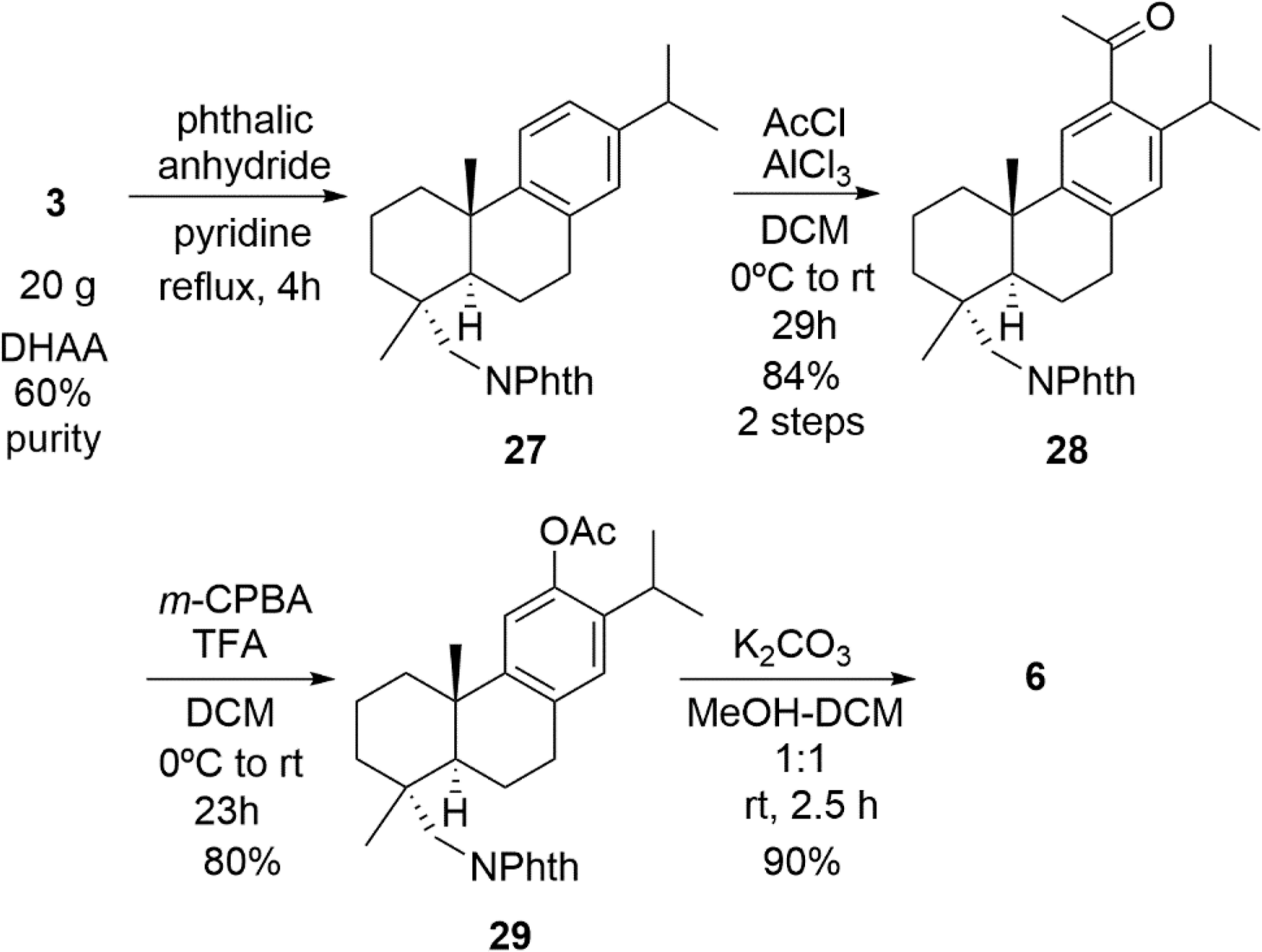
Optimized Synthesis of Antiviral Compound **6**, Starting from DHAA (ca. 60%)

The described procedure in multigram scale for the preparation of antiviral compound **6** includes four synthetic steps (Scheme 2):^22^ (a) preparation of intermediate phthalimide **27**; (b) transformation of **27** into the acetyl derivative **28** by Friedel–Crafts acylation; (c) Baeyer–Villiger oxidation of **28** to give **29**; (d) synthesis of phenol **6** by methanolysis of **29**. However, in the course of the development of further studies of molecule **6**, several problems were found in the original sequence, and, therefore, we have optimized this four-step sequence (Scheme 2).

Following that report, Siegel and co-workers reported in 2013 a quicker route with similar yields but in smaller scale,^26^ using phthaloyl peroxide for the direct hydroxylation of **27**, which seems not useful for scaling-up because of the use of expensive hexafluoro-2-propanol as solvent and the requirement of synthesizing the peroxide, as well as the safety issues for using peroxides in large scale. In 2017, the group of Csuk and co-workers did some modifications to the original method but also in small scale.^27^ For example, they used CH_2_Cl_2_ as solvent for the Friedel–Crafts reaction instead of 1,2-dichloroethane and increased the equivalents of reagents to reduce the reaction time. Also, they modified the conditions of the methanolysis using a higher amount of water as cosolvent.

**Scheme 3:**
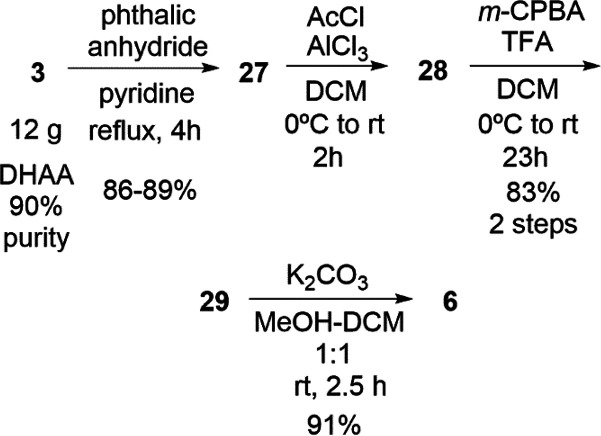
Synthesis of Antiviral Compound **6**, Starting from DHAA (ca. 90%)

Next, we describe in more detail the improved synthetic route for the multigram-scale synthesis of compound **6** (Schemes 2 and 3). In the previously reported condensation step of amine **3** with phthalic anhydride (4 equiv), pyridine was used as solvent.^22^ We evaluated the use of glacial acetic acid, more benign than pyridine as solvent, using **3** as starting material (>90% purity) in 2 g scale, but the resulting yield was lower (74%). For this reason, we kept the original conditions for higher yield, originally 96% starting from **3** (30 g, ca. 65%).^22^ We investigated the use of **3** from different sellers, including the purification of commercially available **3** (ca. 60%) by crystallization of the corresponding acetate salt.^28^ As a result, the overall yield of the synthetic sequence barely changes; however, the purification step of intermediates depends on the purity of starting material **3**.

In the reported Friedel–Crafts reaction of **27**, we kept essentially the same conditions as previously reported,^22^ but changed the solvent to CH_2_Cl_2_ and the workup. Under the conditions reported by Csuk and co-workers,^27^ the Friedel–Crafts reaction of **27** (15 g) obtained from (+)-dehydroabietylamine **3** finished in only 2 h, giving **28** pure enough for the next step, but obviously using double the reagents (Scheme 3).

As for the Baeyer–Villiger reaction of **28**, we used the same conditions for the reaction,^22^ but changed the workup. The acetate removal reaction of **29** proved to be a sensitive reaction, resulting mostly in recovering unreacted acetate.^27^ The reaction was performed in CH_2_Cl_2_–MeOH (1:1) with a stronger base (K_2_CO_3_, 5 equiv). The workup was also modified. Under these new conditions, the reaction was completed in about 3 h, giving 90–93% yield of pure phenol after chromatography with *n*-hexane–EtOAc (7:3). We attempted the direct acetoxylation of **27** as reported recently for similar aromatic systems, giving nitration products instead.^29^

### Antiviral Evaluation

The synthesized compounds **5**, **6**, and **12**–**26** (Scheme 1) were evaluated for antiviral activity against the viruses HHV-1 (*human Alpha herpesvirus type* 1), CHIKV (*Chikungunya virus*), ZIKV (*Zika virus*), and DENV-2 (*Dengue virus type* 2) (Table 1). In the primary screening test for evaluation of broad-spectrum antiviral activity by the end-point titration technique (EPTT),^30^ most of the compounds were active against CHIKV and HHV-1 viruses, but few were active against DENV-2 virus. In the EPTT assay, the lowest concentration with the highest reduction factor (*R_f_*) value was chosen (*Rf* is the ratio of the virus titer in the absence over virus titer in the presence of the compound). According to the parameters established by Vlietinck et al. (1995),^30^ a relevant antiviral activity of a purified natural product is one whose *Rf* of viral titer is 10^2^ (this means that it reduced the viral titer two logarithmic units).

**Table 1:** Antiviral Activity and Cytotoxicity of Ferruginol (**5**), Its C-18 Phthalimide Analogue **6**, Dehydroabietic Acid Derivatives **12**–**14**, and Ferruginol Analogues **15**–**26**

			ZIKV				DENV-2				HHV-1, 29R strain		CHIKV	
	Vero-E6^a^	BHK-21^b^	Vero E6 10TCID_50_^c^		Vero E6 PFU/well:^f^ 150 ± 25		Vero E6 10TCID_50_^c^		BHK21 PFU/well:^f^ 150 ± 25		Vero E6 10TCID_50_^c^		Vero E6 10TCID_50_^c^	
compound	CC_100_^g^ (μg/mL)72h	CC_100_^g^(μg/mL)6 days	inhibition ECytop	(μg/mL)^e^	% inhibition	(μg/mL)^e^	inhibition ECytop	(μg/mL)^e^	% inhibition	(μg/mL)^e^	*R_f_*^d^	(μg/mL)^e^	*R_f_*^d^	(μg/mL)^e^
**5**	>50	>50	++	12.5	38 ± 15	12.5	++	25	28 ± 10	12.5	–	NA	10^2.0^	25
**6**	>50	>50	++	6.2	52 ± 13	3.1	++	25	91 ± 7	12.5	10^2.0^	12.5	10^2.0^	6.2
**12**	>50	50	+	6.2	25 ± 10	12.5	–	NA	–	–	10^1.0^	25	–	NA
13*	50	50	++	12.5	19 ± 12	12.5	+	25	–	NA	10^1.0^	25	10^1.0^	12.5
**14**	>50	>50	++	12.5	37 ± 15	12.5	++	25	30 ± 10	12.5	—-	NA	10^2.0^	25
**15a**	>50	25	–	NA	–	–	–	NA	–	–	10^1.0^	12.5	10^1.0^	25
**15b**	>50	50	++	25	29 ± 14	25	++	25	68 ± 20	25	10^1.0^	25	10^1.0^	25
**16a**	50	50	+	12.5	22 ± 13	12.5	–	NA	–	–	10^1.5^	12.5	10^2.0^	25
**16b**	25	25	+	12.5	29 ± 9	12.5	+	12.5	–	NA	–	NA	10^1.0^	25
**17a**	25	25	–	NA	–	–	–	NA	–	–	10^1.0^	25	–	NA
**17b**	50	25	+	25	–	NA	–	NA	–	–	–	NA	10^1.5^	25
**18a**	50	25	–	NA	–	–	–	NA	–	–	10^1.5^	12.5	10^1.5^	25
**18b**	>50	50	++	25	48 ± 12	12.5	++	25	51 ± 8	12.5	–	NA	10^2.0^	12.5
**19a**	25	12.5	–	NA	–	–	–	NA	–	–	10^1.0^	12.5	10^1.0^	25
**19b**	>50	>50	+	25	27 ± 17	25	++	25	35 ± 10	25	10^1.0^	25	10^1.0^	25
20a*	50	50	++	25	19 ± 11	12.5	++	25	25 ± 10	3.1	10^1.0^	12.5	10^2.0^	12.5
**20b**	25	12.5	+	12.5	–	NA	–	NA	–	–	10^1.5^	12.5	10^2.0^	25
**21a**	25	12.5	++	12.5	27 ± 12	12.5	+	12.5	–	NA	10^1.5^	12.5	10^2.0^	25
**21b**	>25	25	–	NA	–	–	–	NA	–	–	10^1.5^	25	10^1.5^	25
**22a**	50	25	–	NA	–	–	–	NA	–	–	10^1.0^	25	10^1.0^	25
**22b**	25	12.5	–	NA	–	–	+	12.5	–	NA	–	NA	10^1.0^	12.5
**23a**	>50	50	–	NA	–	–	–	NA	–	–	10^1.0^	25	10^1.0^	25
**23b**	25	12.5	–	NA	–	–	–	NA	–	–	–	NA	–	NA
**24**	>25	>25	++	12.5	51 ± 15	12.5	++	25	48 ± 9	3.1	10^1.5^	12.5	10^2.0^	12.5
**25**	>25	>25	++	25	20 ± 09	12.5	–	NA	–	–	–	NA	10^1.5^	25
**26**	>25	>25	+	12.5	–	–	–	NA	–	–	–	NA	10^1.5^	12.5
**DS**	>50	nd	–	nd	–	nd	–	nd	–	nd	10^2.0^	5	–	NA
**H**	>100 U.I	nd	–	nd	–	nd	–	nd	–	nd	10^3.0^	10 U.I.	10^1.0^	10 U.I.
**A**	nd	nd	–	nd	–	nd	–	nd	–	nd	10^2.0^	1.5	nd	nd
**R**	>160	>160	++	40	55 ± 18	20	++	40	75 ± 23	5	10^1.0^	10	10^1.5^	10

a Vero-E6 (African green monkey kidney, *Cercopithecus aethiops*, ATCC CCL-81) cells.

b BHK-21 (baby hamster kidney fibroblasts, *Mesocricetus auratus*, ATCC CCL-10) cells.

c 10 TCID_50_: 10 cell culture infectious dose 50 percent.

d *R_f_*: reduction factor of the viral titer.

e Nontoxic concentration that showed higher viral reduction factor.

f Viruses were quantified by PFU titration (PFU/well), and the infection control was of 150 ± 20 PFU.

g CC_100_: 100% cytotoxic concentrations (μg/mL). Inhibition ECytop: inhibitory cytopathic effect compared to the infection control; a cross (+) was determined for a weak protective effect, and two crosses (++) for a protective effect more than 50% of the monolayer. nd: not determined; NA: not active. DS: dextran sulfate; H: heparin; A: acyclovir; R: ribavirin. Three independent experiments in duplicate for each viral serotype and each concentration were carried out. *Cytostatic compounds. – The evaluation was not carried out because it was not active in the primary screening.

In the anti-CHIKV assays, all compounds were active except **12**, **17a**, and **23b**. Compounds **5**, **6**, **14**, **16a**, **18b**, **20a**, **20b**, **21a**, and **24** showed relevant antiviral activity with an *R_f_* of viral titer of 10^2^. It is worth mentioning that this is, to the best of our knowledge, the first time that anti-chikungunya activity is reported for abietane diterpenoids. In particular, compounds **6**, **20a**, **18b**, and **24** were the most active at concentrations below 12.5 μg/mL, all of which share a common phenol moiety. Compounds **18b** and **24** have two structural features in common since they have only two functional groups in the carbon skeleton: (1) −OH (phenol) and (2) −CO_2_Me. Ferruginol analogue **6** was the most active, having the hydroxyl group at C-12 and the other functional group at C-18, particularly, a phthalimide (−CH_2_NPhth) moiety. Compound **20a** was cytostatic; therefore, it does not allow later to calculate a selectivity index (SI, antiviral selectivity index values (CC_50_/EC_50_)). Also, compound **6** was the only one that showed an *R_f_* of 10^2^ against the HHV-1 viral model of the DNA genome. Compounds **5**, **14**, **16b**, **17b**, **18b**, **22b**, **23b**, **25**, and **26** were not active for HHV-1, indicating partially that compounds having a methyl ester group at C-19 do not have anti-herpes type 1 activity.

Following our research, the EPTT assay showed that Zika virus was more sensitive to cytopathic effect inhibition exerted by the tested compounds than DENV-2 virus. Compounds **5**, **6**, **13**, **14**, **15b**, **18b**, **20a**, **21a**, **24**, and **25** showed a protective effect of more than 50% of the monolayer (++); but only compounds **5**, **6**, **13**, **14**, **21a**, and **24** showed cytopathic effect inhibition at concentrations below 12.5 μg/mL. Again, compound **6** was the most active at concentrations of 6.2 μg/mL, while for DENV-2 virus, only concentrations above 25 μg/mL of compounds **5**, **6**, **14**, **15b**, **18b**, **19b**, **20a**, and **24** showed a protective effect of more than 50% (++). This primary screening test (EPTT) to evaluate antiviral activity between flaviviruses is not sensitive enough to carry out structure–activity relationship studies, since it is only a qualitative approximation about cytopathic effect inhibition. Thus, the compounds that showed a protective factor of more than 50% were evaluated by the quantitative technique of plaque-forming units (PFU). The concentrations evaluated were also the same EPTT assay dilutions/4, and the lowest concentration with the highest percentage of reduced PFU with respect to the control (150 PFU) was chosen. The order of activity of the compounds at the concentration of 12.5 μg/mL against the Zika virus was **24**, **18b** > **14**, **5** > **15b**, **16b**, **16a**, **19b**, **21a**, **12** > **20a**, **25**, **13**. Compound **6** was the most active at concentrations of 3.1 μg/mL (ca. 7.2 μM, nontoxic concentration that showed a higher viral reduction factor).

In the anti-DENV-2 assays, the order of activity of the compounds at the concentration of 12.5 μg/mL was **6** > **18b** > **14**, **5**. Compound **24** was the most active at a concentration of 3.1 μg/mL (ca. 9.3 μM) for DENV-2 followed by compound **20a**; however, the latter was cytostatic.

Therefore, for antiflaviviruses (ZIKV and DENV-2) activity, some of the most active compounds (**18b** and **24**) share two functional groups on the abietane carbon skeleton: (1) −OH and (2) −CO_2_Me.

In previous results published by our group, the anti-herpetic and anti-DENV activities have been evaluated for compound **6**. This molecule has its antiviral effect mainly in postinfection stages: in DENV-2 with 50% effective antiviral concentration (EC_50_) of 1.4 μM and for herpes virus type 2 (HHV-2) of 19.2 μM,^8^ as well as for a Brazilian Zika (clinical isolate, IMT17) virus strain of 7.7 μM.^31^ Therefore, since the compounds ferruginol (**5**), ferruginol analogue **6**, **14**, **18b**, and **24** presented better results in the preliminary screening, the broad-spectrum antiviral activity was evaluated in postinfection stages. Thus, the 50% effective antiviral concentration (EC_50_) was determined against ZIKV, DENV-2, and CHIKV. Only compound **6** had a dose-dependent effect in postinfection stages. For the Zika_459148 virus strain the increased viral infectivity was achieved through several passages in the Vero-E6 cell line. From the 15th pass, titers greater than 1 × 10^7^ PFU/mL were obtained. Compound **6** gave an anti-Zika_459148 virus activity EC_50_ value of 6.3 ± 2.7 μM for treatment of 72 h (CC_50_ = 192 μM, SI = 30), while the control ribavirin gave an EC_50_ value of 83 ± 3.9 μM (CC_50_ > 160 μM); moreover, we found that the EC_50_ for another ZIKV strain (COL345Si) was 5.3 ± 2.9 μM for a treatment of 6 days (CC_50_ = 80.8 μM, SI = 15). The difference in the incubation time (6 days) of the last mosquito-viral strain corresponds to the time required to see lysis plaques on mammalian cells of a viral strain in which their viral infectivity was not increased through several passages. The EC_50_ value of the control during 6 days for ribavirin was 101 ± 3.7 μM (CC_50_ > 160 μM). It is noteworthy that compound **6** was even active in postinfection stages against endemic chikungunya virus of Colombian origin with EC_50_= 9.8 ± 2.5 μM (SI = 19.6).

**Figure 1:**
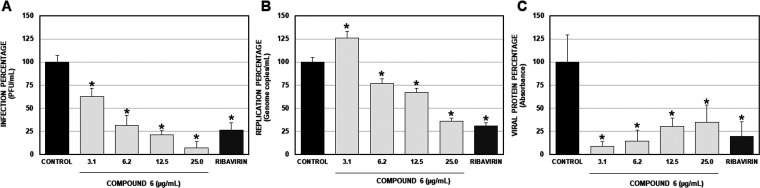
Antiviral effects on compound **6** on cultures treated and posteriorly infected with ZIKV/Col. (A) Effect on release of viral infectious particles. Infection percentage calculated according to the results obtained by plaque assay (PFU/mL) of the supernatants collected. (B) Effect on intracellular genomic copies. The replication percentage was calculated according to the results obtained by qRT-PCR of monolayers infected. (C) Effect on intracellular viral protein. Viral protein percentage was calculated according to the results obtained by cell-ELISA on monolayers infected. The asterisks indicate statistically significant differences with respect to the control without compound (*p < 0.05; Student’s *t* test), and error bars indicate the standard error of the mean; *n* = 6. In all cases ribavirin (100 μM) was used as the positive control of inhibition.

The antiviral effect of compound **6** was further confirmed, using other viral strain (ZIKV/Col), by three different methodologies: titration by plaque assay, RT-qPCR, and cell-ELISA (Figure 1). In each case, the percentage of infection was calculated based on the untreated control (100% infection) as per the units of measure of each technique. In all cases ribavirin (100 μM) was used as an inhibition positive control. In the supernatants of cultures of Vero cells infected with ZIKV/Col and post-treated with four concentrations (3.1, 6.2, 12.5, and 25.0 μg/mL), the number of viral infectious particles was quantified by plaque assay.^32^ We found that all concentrations of compound **6** significantly inhibited the production of infectious viral particles of ZIKV/Col. In this sense, the percentages of infection were 62.7%, 31.3%, 20.9%, and 7.0%, respectively, compared with the untreated control (Figure 1A) (EC_50_ = 9.9 μM, SI = 32.6). On the other hand, compound **6** significantly inhibited the replication of viral genome determined by qRT-PCR^33^ in the cultures treated with 6.2, 12.5, and 25.0 μg/mL, but in cultures treated with 3.1 μg/mL the number of genome copies/mL was increased significantly (125.9%) (Figure 1B). It is not surprising to find accumulation of viral genomes inside the cells in the presence of antiviral compounds;^34^ in fact, a block in the viral assembly can produce an increase in viral genome with a slight reduction in infectious viral particles, as has been reported previously in another flavivirus model.^32^

Finally, we found that all concentrations evaluated significantly inhibited the viral protein production quantified by cell-ELISA (percentages of infection below 50%) (Figure 1C).^35^

To approach a possible molecular explanation of the broad-spectrum antiviral action in postinfection stages of compound **6**, a molecular docking analysis was performed using Autodock Vina software^36^ to predict the interaction between abietanes **5**, **6**, and **24** and viral targets (NS5, NS3, nsP2, among others) or cellular targets involved in both tubulin and actin polymerization or depolymerization pathways (as well as the different constituents of the cytoskeleton) (see Supporting InformationTable S1; Table 2 for lead compound **6**). The latter cell targets were chosen because the cytoskeletal components such as microtubules and actin microfilaments are required for most viruses such as flavivirus,^37^ herpesvirus and retrovirus,^38^ and coronavirus,^39^ among others, for their replication cycle. The best interaction and correlation with the antiviral values of the three abietanes was with G-actin; however, interactions with the NS5 viral flavivirus proteins is not ruled out, as it was NS5 rdrp for ZIKV and DENV, respectively. With this *in silico* analysis, it can be postulated that both cell and viral targets are involved in the mechanism of action of these three compounds and that organelles made up of G-actins are required for viral production, mainly for flavivirus, as has been shown by other authors. Further experiments are needed, however, to confirm this hypothesis.

**Table 2:**
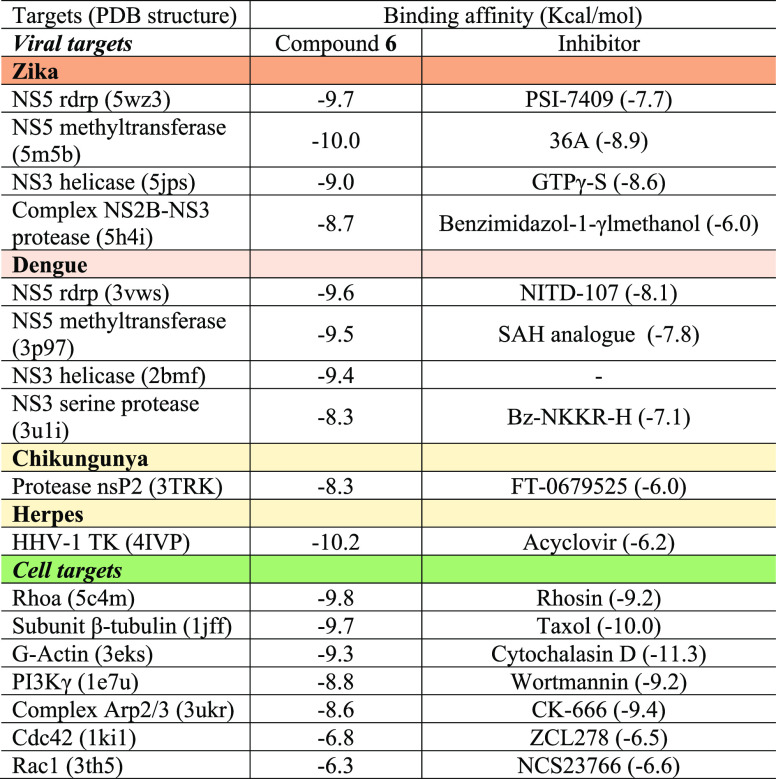
Binding Energy Affinities (kcal/mol) of Compound **6** and Control Inhibitors for Several Viral and Cellular Targets

In summary, an extended antiviral study of a number of semisynthetic abietanes confirms that both ferruginol (**5**) and its analogue **6** are both broad-spectrum antivirals with interesting biological properties, as are some tested ferruginol analogues. A basic structure–activity trend for broad-spectrum antiviral activity that can be deduced is a C-12 hydroxy group with a C-18 phthalimide group (compound **6**) and a C-14 hydroxy group with a C-18 methyl ester (compound **24**), while C-12 acetyl or acetoxy as well as C-7 carbonyl groups led to less active compounds independently of the substituent at C-18 or C-19. Also, a synthetic route has been optimized in multigram scale for the preparation of antiviral ferruginol analogue **6** (60% overall yield, four steps), starting from commercially available (+)-dehydroabietylamine **3**. The sequence has been optimized to use only three reaction solvents (pyridine, CH_2_Cl_2_, and MeOH), and workup and purification methods, and even reaction conditions, have been changed to solve some problems found in the original procedures. In conclusion, it has been demonstrated that ferruginol analogues present relevant antiviral activity.

## Experimental Section

### General Experimental Procedures

Optical rotations were measured using a 10 cm cell in a Jasco P-2000 polarimeter in dichloromethane unless otherwise stated. NMR spectra were recorded on a 400 MHz spectrometer. All spectra were recorded in CDCl_3_ as solvent unless otherwise stated. Reactions were monitored by TLC using Merck silica gel 60 F254 (0.25 mm thick) plates. Compounds on TLC plates were detected under UV light at 254 nm and visualized by immersion in a 10% sulfuric acid solution and heating with a heat gun. Purifications were performed by flash chromatography on Merck silica gel (230–400 mesh). Commercial reagent grade solvents and chemicals were used as purchased unless otherwise noted. Combined organic extracts were washed with brine, dried over anhydrous MgSO4, filtered, and concentrated under reduced pressure.

The starting material, dehydroabietylamine (**3**, ca. 60%), was purchased from Aldrich and that of purity >90% from TCI Europe. The carbon numbering of all synthetic compounds corresponds to that of natural products.

### Materials

All compounds prepared in this work display spectroscopic data in agreement with the reported data.^8,15,23−25^ Complete details of the optimized preparation of **6** are given in the Supporting Information. The purity of compounds was 95% or higher.

### Antiviral Activity

Complete details of viruses and assays are given in the Supporting Information.

### Molecular Docking

Details are given in the Supporting Information.

## Supporting Information Available

Complete experimental details for the optimized resynthesis of compounds **5** and **6** and for all the biological assays and docking modeling; copies of ^1^H and ^13^C spectra for intermediates and final compound **6** and ^1^H spectrum of the mixture of nitro derivatives (PDF)
