# Supplementary material for: Antiviral Profiling of C-18- or C-19-Functionalized Semisynthetic Abietane Diterpenoids
Source: J Nat Prod. 2022 Aug 15;85(8):2044–51. doi: 10.1021/acs.jnatprod.2c00464 (PMC9425435; doi:10.1021/acs.jnatprod.2c00464)
Supplement: Supplementary file 1 — np2c00464_si_001.pdf [file np2c00464_si_001.pdf]

Electronic Supporting Information

Antiviral Profiling of C18- or C19-Functionalized Semisynthetic Abietane Diterpenoids

Miguel A. González-Cardenete[†,*] *Damir Hamulić,*[†] *Francisco J. Miquel-Leal,*[†] *Natalia González-Zapata,*[†] *Orlando J. Jimenez-Jarava,*[§] *Yaneth M. Brand,*[§] *Laura C. Restrepo-Mendez,*[ǂ] *Marlen Martinez-Gutierrez,* [ǂ,‡] *Liliana A. Betancur-Galvis,* [§] *and Maria L. Marín,*[†]

[†] Instituto de Tecnología Química (UPV-CSIC), Universitat Politècnica de Valencia-Consejo Superior de Investigaciones Científicas, Avda de los Naranjos s/n, 46022 Valencia, Spain

[§] Grupo de Investigaciones Dermatológicas, Instituto de Investigaciones Médicas, Facultad de Medicina, Universidad de Antioquia, 050010 Medellín, Colombia.

[ǂ] Grupo de Investigación en Ciencias Animales-GRICA. Universidad Cooperativa de Colombia. Bucaramanga, Colombia.

[‡] Línea de descubrimiento y evaluación de compuestos antivirales. Grupo de Investigación en Microbiología Básica y Aplicada (MICROBA), Escuela de Microbiología, Universidad de Antioquia, 050010, Medellín, Colombia.

*Correspondence: migoncar@itq.upv.es

Number of pages: 33

EXPERIMENTAL SECTION

I) Synthesis

*Synthesis of N-phthaloyldehydroabietylamine (**27**).*

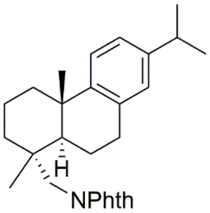

27

Adapted from Malkowsky and co-workers.[1] (+)-Dehydroabietylamine **3** (ca. 60% aldrich, 20 g, ca. 42 mmol) was dissolved in pyridine (90 mL) and phthalic anhydride (24,88 g, 168 mmol, 4 equiv.) was added at rt. The reaction mixture was heated at reflux (in a heating block with hot plate at 135 °C) and stirring at 400 rpm for 4 h. After cooling at rt, the mixture was poured onto a beaker with cold water (300 mL) and was extracted with diethyl ether (100 mL and 2 × 80 mL). The combined organic phases were washed with 10% aq. HCl (2 × 80 mL), H_2O (2 × 50 mL) and brine (50 mL), dried ($MgSO_4$) under stirring overnight. Next day, the extract was filtered and concentrated to give 31.5 g of pale yellow oil, which could not be induced to crystallize with absolute EtOH. Then, the crude was chromatographed on silica (ca. 200 g) eluting with *n*-hexane-EtOAc (9:1) to give 24.9 g of phthalimide **27** as a yellowish semisolid, which had ^{1}H and ^{13}C NMR data in agreement with those reported[1] and showing some unidentified minor impurities, which do not affect next step: ^{1}H NMR (400 MHz) δ 7.82-7.80 (2H, m), 7.70-7.68 (2H, m), 7.12 (1H, d, *J* = 8.0), 6.95 (1H, dd, *J* = 8.0, 2.0), 6.91 (1H, d, *J* = 2.0), 3.68 (1H, d, *J* = 13.6), 3.51 (1H, d, *J* = 13.6), 3.04-2.97 (2H, m), 2.81 (1H, sept., *J* = 6.8), 2.27-2.22 (2H, m), 1.80-1.25 (7H, m), 1.23 (3H, d, *J* = 6.8), 1.21 (6H, d, *J* = 6.8), 1.06 (3H, s); ^{13}C NMR (100 MHz) δ_C 2 × 169.3 (s), 147.2 (s), 145.5 (s), 135.0 (s), 2 × 133.9 (d), 132.0 (s), 127.0 (d), 123.8 (d), 123.7 (d), 2 × 123.2 (d), 48.9

(t), 45.1 (d), 39.4 (s), 38.1 (t), 37.6 (s), 36.9 (t), 33.4 (d), 30.1 (t), 25.9 (q), 24.0 (q), 23.9 (q), 19.4 (t), 19.1 (q), 18.5 (t); HRMS (ESI) m/z 438.2463 $[M+ Na]^+$, calcd for $C_{28}H_{33}NO_2Na$: 438.2409.

*Attempt of obtaining **27** in AcOH as solvent instead of pyridine:*

A mixture of (+)-Dehydroabietylamine **3** (ca. 90% TCI Europe, 2.0 g, ca. 7 mmol) and phthalic anhydride (4,0 g, 28 mmol, 4 equiv.) was suspended in glacial acetic acid (15 mL) and heated at reflux (in a heating block with hot plate at 135 °C) and for 4 h. After cooling at rt overnight, ice and water and diethyl ether (10 mL) were added and the mixture was extracted with diethyl ether (3 × 15 mL). The combined organic phases were washed with saturated aq. $NaHCO_3$ (30 mL + 2 × 10 mL), H_2O (1 × 10 mL) and brine (10 mL), dried ($MgSO_4$) and concentrated to give 3.4 g of pale semisolid. Then, the crude was chromatographed on silica eluting with *n*-hexane-EtOAc (9:1) to give 2.15 g of phthalimide **27** as a yellowish semisolid (74%), which had ^1H and ^{13}C NMR data in agreement with those reported.[1]

*Synthesis of 12-acetyl-N-phthaloyldehydroabietylamine (**28**).*

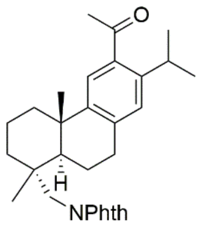

28

Adapted from Malkowsky and co-workers.[1] A solution of compound **27** from previous step (24.9 g, ca. 42 mmol) in DCM (300 mL) was cooled in an ice-bath and AcCl (10.45 mL, 11.53 g, 147 mmol, 3.5 equiv.) was added followed by $AlCl_3$ (16.8 g, 126 mmol, 3.0 equiv.). The reaction mixture became from yellowish to dark brown and stirred for 20 min. Then, the ice-bath was

removed and the reaction was stirred for 29 h at rt. After this time, the resulting brownish-red solution was cooled in an ice-bath and quenched dropwise in a beaker with saturated aq. $NaHCO_3$ (100 mL) (Gas evolution!). The mixture was poured onto saturated aq. $NaHCO_3$ (200 mL) in a 1L separation funnel and the phases were separated. Care must be taken with continuous opening of the tap of the funnel to avoid CO_2 pressure. The aqueous phase was extracted with DCM (2 × 100 mL). The combined organic phases were washed with H_2O (100 mL) and brine (50 mL), dried ($MgSO_4$) under stirring overnight. Next day, the extract was filtered and concentrated to give 29.2 g of yellowish brown semi-solid which was crystallized with EtOH (90 mL) overnight. The resulting greenish solid was filtered off under vacuum and washed with cold EtOH (60 mL) and dried under vacuum to give 16.1 g of acetyl derivative **28** as a pale greenish solid (84%, two steps), which had ^{1}H and ^{13}C NMR data in agreement with those reported.[1] From the mother liquor were recovered, after chromatography on silica eluting with *n*-hexane-EtOAc (8:2), additional 4.8 g of product as a yellow solid. ^{1}H NMR (300 MHz) δ 7.83-7.80 (2H, m), 7.71-7.68 (2H, m), 7.35 (1H, s), 7.07 (1H, s), 3.69 (1H, d, J = 13.8), 3.50 (1H, d, J = 13.8), 3.45 (1H, sept., J = 6.9), 3.02-2.99 (2H, m), 2.51 (3H, s), 2.28-2.23 (2H, m), 1.85-1.30 (7H, m), 1.23 (3H, s), 1.21 (3H, d, J = 6.9), 1.18 (3H, d, J = 6.9), 1.07 (3H, s); ^{13}C NMR (100 MHz) δ_C 203.3 (s), 2 × 169.3 (s), 146.9 (s), 144.8 (s), 139.2 (s), 136.1 (s), 2 × 133.9 (d), 132.0 (s), 127.1 (d), 124.1 (d), 2 × 123.2 (d), 48.7 (t), 44.9 (d), 39.4 (s), 38.0 (t), 37.5 (s), 36.8 (t), 30.4 (q), 30.1 (t), 28.6 (d), 25.8 (q), 24.2 (q), 24.1 (q), 19.2 (t), 19.1 (q), 18.4 (t); HRMS (ESI) m/z 480.0443 $[M+ Na]^+$, calcd for $C_{30}H_{35}NO_3Na$: 480.2515.

*Synthesis of 12-acetoxy-N-phthaloyldehydroabietylamine (**29**).*

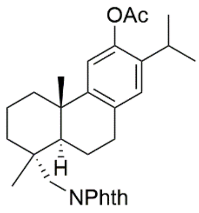

29

Adapted from Malkowsky and co-workers.[1] Compound **28** (20.7 g, 45.7 mmol) and *meta*-chloroperbenzoic acid (MCPBA, 27.3 g, 118.9 mmol, 2.6 equiv.) were dissolved in DCM (125 mL) and cooled in an ice-bath. Then, trifluoroacetic acid (3.5 mL, 5.2 g, 45.7 mmol, 1.0 equiv.) was added dropwise and the mixture was stirred for 20 min. before allowed to warm to rt and stirring continued for 23h. Next day, the reaction mixture was diluted with DCM (80 mL) and quenched with 10% aq. Na_2SO_3 (120 mL). Phases were separated in a 1L separation funnel and the aqueous phase was extracted with DCM (60 mL). The combined organic phases were washed with H_2O (100 mL + 10 mL of brine), 50% saturated aq. $NaHCO_3$ (2 × 125 mL), and brine (100 mL), dried over $MgSO_4$ under stirring for 30 min., filtered and concentrated to give a crude of 23 g as a pale oil. The crude was chromatographed on silica eluting with *n*-hexane-EtOAc (7:3) to give 16.9 g (80%) of acetate **29** as a yellow foam, which had ^{1}H and ^{13}C NMR data in agreement with those reported:[1] ^{1}H NMR (400 MHz) δ 7.83-7.80 (2H, m), 7.71-7.68 (2H, m), 6.96 (1H, s), 6.77 (1H, s), 3.69 (1H, d, *J* = 13.6), 3.48 (1H, d, *J* = 13.8), 2.98-2.95 (2H, m), 2.89 (1H, sept., *J* = 6.8), 2.28 (3H, s), 2.28 (1H, m), 2.11 (1H, br d, *J* = 12.8), 1.85-1.30 (7H, m), 1.22 (3H, s), 1.18 (3H, d, *J* = 6.8), 1.15 (3H, d, *J* = 6.9), 1.05 (3H, s); ^{13}C NMR (100 MHz) δ_C 170.0 (s), 2 × 169.3 (s), 148.4 (s), 145.9 (s), 136.7 (s), 2 × 133.9 (d), 133.2 (s), 132.0 (s), 127.1 (d), 2 × 123.2 (d), 117.5 (d), 48.7 (t), 44.5 (d), 39.4 (s), 38.0 (t), 37.5 (s), 36.8 (t), 29.5 (t), 27.1 (d), 25.8 (q), 23.0 (q), 22.9

(q), 20.9 (q), 19.3 (t), 19.2 (q), 18.4 (t); HRMS (ESI) m/z 496.0274 $[M+ Na]^+$, calcd for $C_{30}H_{35}NO_4Na$: 496.2464.

*Synthesis of 12-hydroxy-N-phthaloyldehydroabietylamine or 18-(Phthalimid-2-yl)ferruginol (**6**).*

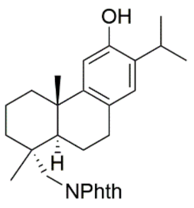

6

Compound **29** (10.8 g, 22.8 mmol) was dissolved in DCM (80 mL) and absolute MeOH (80 mL). Then, K_2CO_3 (15.8 g, 114.2 mmol, 5.0 equiv.) was added in portions under continuous stirring at rt and the heterogeneous yellow mixture became reddish-brown. After 2.5h, monitored by TLC (eluted twice with *n*-hexane-EtOAc (8:2)), the mixture was filtered under vacuum in a sintered or Büchner funnel and the solid washed with DCM (60 mL + 20 mL). Then, the filtrate was acidified with 10% aq. HCl (ca. 10 mL) until a colour change to yellow was observed and pH= 1-2. The solution was washed with brine (40 mL) which was re-extracted with additional 20 mL of DCM. The combined organic phases were dried over $MgSO_4$ under stirring overnight. Next day, it was filtered and concentrated to give a crude of 9.6 g as a yellow solid. The crude was chromatographed on silica eluting with *n*-hexane-EtOAc (7:3) to give 8.94 g (90%) of phenol **6** as a yellow foam, which had ^1H and ^{13}C NMR, and specific optical rotation ($[\alpha]^{23}_D$ –31.4 (c 0.7, DCM) data in agreement with those reported:[1] ^1H NMR (400 MHz) δ 7.82-7.80 (2H, m), 7.70-7.68 (2H, m), 6.86 (1H, s), 6.59 (1H, s), 4.59 (1H, br s), 3.67 (1H, d, J = 14.0), 3.50 (1H, d, J = 14.0), 3.09 (1H, sept., J = 6.8), 2.93-2.90 (2H, m), 2.25-2.21 (1H, m), 2.12 (1H, br d, J = 12.8), 1.83 (1H, m), 1.73-1.61 (3H, m), 1.51-1.48 (1H, m), 1.38-1.25 (2H, s), 1.23 (3H, d, J = 6.8), 1.21 (3H, d, J = 6.8),

1.21 (3H, s), 1.04 (3H, s); ^{13}C NMR (100 MHz) δ_C 2 × 169.3 (s), 150.7 (s), 148.2 (s), 2 × 133.9 (d), 132.0 (s), 131.6 (s), 127.2 (s), 126.8 (d), 2 × 123.2 (d), 110.5 (d), 48.9 (t), 45.2 (d), 39.4 (s), 38.1 (t), 37.5 (s), 36.9 (t), 29.3 (t), 26.8 (d), 25.7 (q), 22.7 (q), 22.6 (q), 19.5 (t), 19.1 (q), 18.5 (t); HRMS (ESI) m/z 454.0478 $[M+ Na]^+$, calcd for $C_{28}H_{33}NO_3Na$: 454.2358. Anal. calcd. for $C_{28}H_{33}NO_3$: C, 77.9; H, 7.7; N, 3.2. Found: C, 77.6; H, 7.8; N, 3.1.

*Synthesis of (+)-ferruginol (**5**).*

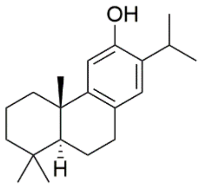

5

Adapted from González and Perez-Guaita, 2012.[2] 12-Hydroxydehydroabietylamine (103 mg, 0.34 mmol) is dissolved in 2.5 M NaOH (0.7 mL) and ethanol (1 mL) and cooled to 0 ºC. Then, hydroxylamine O-sulfonic acid (HOS 97%, 75 mg, 0.66 mmol, 2 equiv.) is added. After stirring for 20 min, additional 2.5 M NaOH (0.3 mL) and ethanol (0.3 mL) were added followed by 60 mg (1.5 equiv.) of additional HOS and the mixture was allowed to warm to rt. After 3h 30 min, additional HOS (100 mg, 0.88 mmol, 2.6 equiv.) were added and stirring continued overnight. After 24h, additional 2.5 M NaOH (0.3 mL) and HOS (67 mg, 0.60 mmol, 1.7 equiv.) were added and 3h later, the basic solution was neutralized with concentrated 10% aqueous HCl (ca. 0.8 mL) dropwise, diluted with ethyl acetate (15 mL) and washed with brine, dried and concentrated. The resulting residue (115 mg, pale semisolid) was chromatographed, dissolving it with eluent and some drops of ethyl acetate, on silica eluting with *n*-hexane-ethyl acetate 8:2 to give 62 mg (63%) of the desired compound **5** as a pale oil, which had ^{1}H and ^{13}C NMR data in agreement with those reported:[2] ^{1}H NMR (400 MHz) δ 6.84 (1H, s), 6.64 (1H, s), 3.12 (1H, sept., J = 6.8), 2.89-2.75

(2H, m), 2.16 (1H, m), 1.89-1.84 (1H, m), 1.75-1.57 (3H, m), 1.47 (1H, m), 1.41-1.31 (2H, m), 1.24 (6H, d, J = 6.0), 1.18 (3H, s), 0.95 (3H, s), 0.93 (3H, s); ^{13}C NMR (75 MHz) δ_C 150.6 (s), 148.6 (s), 131.5 (s), 127.2 (s), 126.5 (d), 111.0 (d), 50.3 (d), 41.7 (t), 38.8 (t), 37.4 (s), 33.4 (s), 33.3 (q), 29.7 (t), 26.7 (d), 24.7 (q), 22.7 (q), 22.6 (q), 21.6 (q), 19.3 (t), 19.2 (t); HRMS (ESI) *m/z* 287.2369 $[M+H]^+$, calcd for $C_{20}H_{31}O$: 287.2375. Anal. calcd. for $C_{20}H_{30}O$: C, 83.9; H, 10.6. Found: C, 83.6; H, 10.2.

II) Biological assays

Reagents and compounds

Dulbecco's Modified Eagle's Medium (DMEM), L-glutamine, non-essential amino acids and minimum essential medium vitamin solution, $NaHCO_3$, carboxymethylcellulose sodium salt medium viscosity (CMC) and 3-(4,5-dimethylthiazol-2-yl)-2,5-diphenyl tetrazolium bromide (MTT) were obtained from Sigma-Aldrich Chemical Co. (St. Louis, MO, USA). Fetal bovine serum (FBS) and penicillin/streptomycin were purchased from Invitrogen Life Technologies (Carlsbad, CA, USA). Ribavirin was obtained from Calbiochem (La Jolla, CA, USA). 18-(Phthalimide-2-yl) ferruginol, ferruginol and Ribavirin stock solutions were prepared in dimethyl sulfoxide (DMSO, Sigma, Cream Ridge, NJ, USA) to be evaluated immediately.

Cell culture and viruses

Vero-E6 cells (African green monkey kidney-Cercopithecus aethiops, ATCC CRL- 1586), Vero cells (African green monkey kidney-Cercopithecus aethiops, ATCC: CCL 81 line), BHK-21 cells (Baby hamster kidney fibroblasts - *Mesocricetus auratus*, ATCC CCL-10) and C6/36HT cells *(Aedes albopictus*, ATCC CRL-1660) were maintained in Dulbecco's Modified Eagle's Medium (DMEM) supplemented with 5% of inactivated fetal bovine serum (FBS) (10% in the case of C6/36HT cells), 100 units/mL of penicillin, 100 mg/mL of streptomycin, 100 mg/mL of L-

glutamine, 0.14% $NaHCO_3$, and 1% of each non-essential amino acids and minimum essential medium vitamin solution (choline chloride, D-calcium pantothenate, folic acid, nicotinamide, pyridoxal hydrochloride, riboflavin, thiamine hydrochloride and i-inositol). Vero CCL-81, Vero E6 and BHK-21 cells were incubated at 37 ºC in humidified 5% CO_2 atmosphere and C6/36 HT at 34 ºC in humidified 5% CO_2 atmosphere.

Zika virus_459148 (clinical isolate, Zika_virus_459148_Meta_Colombia_2016/GenBank-MH544701.2) and CHIKV was donated by Virology Group / "Dirección de Redes en Salud Pública" (Instituto Nacional de Salud, Bogotá, DC, Colombia). Its characterization was described by Laiton-Donato et al., 2019.[3] Zika virus COL345Si (isolate from mosquitoes in Sucre, Colombia/GenBank- MH179341.1) was donated by Immunology Group/ Facultad de Medicina, Universidad de Antioquia, Colombia. Viruses stock were produced and titrated in Vero-E6 cells for ZIKV and Vero for CHIKV using the technique plaque assay.

Human Alphaherpesvirus type 1 (HHV-1, 29R strain) was purchased from the Center for Disease Control. HHV-1 was amplified in Vero cells (African Green monkey kidney-*Cercopithecus aethiops*, ATCC CCL-81 line). Virus stocks were titrated in Vero E6 cells (African Green monkey kidney-*Cercopithecus aethiops,* ATCC CRL- 1586) by plaque assay and expressed as plaque forming units (PFU/mL). Dengue virus type 2 (DENV-2 New Guinea strain) was donated by Maria Elena Peñaranda and Eva Harris (Sustainable Sciences Institute and the University of California at Berkeley). The virus was amplified in C6/36HT cells *(Aedes albopictus*, ATCC CRL-1660) and titrated in BHK-21 cells (Baby hamster kidney fibroblasts - *Mesocricetus auratus*, ATCC CCL-10).

End-point titration technique (EPTT).

The technique EPTT (Vlietinck et al., 1995),[4] with few modifications was used. The unit used in the technique EPTT for CHIKV and HHV-1 was one $TCDI_{50}$, that means the dilution of the virus required to obtained 50% lytic effect of the cellular culture in each well in 100 μL of viral suspension ($TCDI_{50}$/0.1mL) in 72h of infection. For ZIKV and DENV-2, the term $TCDI_{50}$ was used to describe the dilution of the virus required to obtained 50% cytopathic effect of the culture also in 72 h of infection. For all four viruses, confluent monolayer Vero-E6 were grown in 96-well flat-bottomed plates (2.0 x 10^3 cells/well), at 37 ºC in humidified 5% CO_2 atmosphere. After 24h of incubation two-fold dilutions of the compounds were prepared in Dulbecco's Modified Eagle's Medium (DMEM) supplemented with 100 units/mL of penicillin, 100 mg/mL of streptomycin, 100 mg/mL of L-glutamine, 0.14% $NaHCO_3$, and 1% of each non-essential amino acids and minimum essential medium vitamin solution (maintenance medium, identical to growth medium except the FBS). Then, viral suspension (10$TCDI_{50}$)/compound mixture (1/1 v/v) in DMEM with 2% FBS supplemented containing carboxymethyl cellulose (CMC) to 0.5% were incubated for 15 min at room temperature and 100 μL added on each well. Compounds were evaluated finally in well at concentrations of 3.1 to 25 μg/mL. Three independent experiments by duplicated for each viral serotype and each concentration were carried out. Controls were included: untreated cells, cells treated with compounds and cells infected with each viral type. The concentration of DMSO in assays was of 0.05% and cellular controls with DMSO at 0.05% were used. Positive controls included: Acyclovir (A), Ribavirin (R), Heparin (H) and Dextran Sulfate (DS). After 72 h of incubation at 37º C in humidified 5% CO_2 atmosphere, the cell monolayers were stained with a solution of 3.5% formaldehyde with 0.2% crystal violet and cytopathic effect (CPE) was observed under inverted microscope. The reduction in virus titer was determined as the Rf of the virus titer, i.e., the ratio of the virus titer in the absence over virus titer in the presence of the compound of

each concentration tested for HHV-1 and CHIKV viruses. According to the parameters established by Vlietinck et al. (1995),[4] a relevant antiviral activity of a purified natural product is one whose reduction factor (Rf) of viral titer is 10^2. This parameter was used in our study. The cytopathic effect caused ZIKV and DENV-2 was defined as the change in the cellular morphology of the infected monolayer in 72 h with respect to uninfected cells control and it was evaluated qualitatively. A cross (+) was determined for a weak protective effect, and two crosses (++) for a protective effect more than 50% of the monolayer.

Plaque reduction assay.

The (positive) qualitative evaluation (++) of the primary antiviral screening (EPTT) for DENV-2 and ZIKA viruses was confirmed by the plaque-forming unit technique (PFU) or Plaque reduction assay, using the same protocol of simultaneous addition of compound and virus as described above; but this time in 48- well plates. Briefly, Vero-E6 or BHK-21 were grown in 48-well flat-bottomed plates (1.0 x 10^4 cells/well in 200 µL), at 37° C in 5% CO_2 humidified atmosphere. After 24h of incubation two-fold dilutions (12.4 to 100 µg/mL) of the compounds were prepared in Dulbecco's Modified Eagle's Medium (DMEM) supplemented. Then, viral suspension (300 PFU)/compound mixture (1/1 v/v) in DMEM with 2% FBS supplemented containing carboxymethyl cellulose (CMC) to 1.0 % were incubated for 15 min at room temperature and 200 µL added on each well. After 72 hours for ZIK virus (Vero-E6 cells) or six days for DENV virus (BHK-21 cells) fixation and staining were performed, as previously described above. Viral plaque counting was performed in 3 independent assays for duplicated to determine the inhibition of plaques formation in percentages (%) with respect to untreated infected control.

Post-infection Stage Evaluation Assay.

Compounds **6**, **14**, **18b** and **24** were evaluated during post-infection stage. Briefly, 2.5 x 10^4 cells/well of Vero-E6 cell for anti- Zika and CHIKV assays and BHK-21 cells for anti-DENV-2 assay were grown in 24-well flat-bottomed plates in 250 μL of Dulbecco's Modified Eagle's Medium (DMEM) supplemented with 5% of inactivated fetal bovine serum (FBS), 100 units/mL of penicillin, 100 mg/mL of streptomycin, 100 mg/mL of L-glutamine, 0.14% $NaHCO_3$, and 1% of each non-essential amino acids and minimum essential medium vitamin solution. After 24 h of incubation at 37 ºC in humidified 5% CO_2 atmosphere, the plates were infected with the virus CHIK, DENV-2, ZIKA COL345Si or ZIKV 459148 at MOI= 1 and incubated for 2 h. Then, the wells were washed with PBS and two fold dilutions of the compounds were added in DMEM supplemented with 2% FBS containing carboxymethyl cellulose (CMC) to 1.5%. Controls were included: untreated cells, cells treated with compounds and cells infected with each viral type. The concentration of DMSO in assays was of 0.05% and cellular controls with DMSO at 0.05% were used. For each assay described here, the Ribavirin, was used as a positive control at concentrations of 20- 160 μM and the compounds were evaluated finally in well at non- cytotoxic concentrations of 3.1 to 25 μM. Finally, after 6 days of incubation for Zika COL345Si and DENV-2 virus, and 72 h for Zika _459148 and CHIK virus; fixation and staining were performed, as previously described for the plaque forming unit (PFU) assay. Viral plaque counting was performed in 3 independent assays to determine the inhibition of plaque formation. The effective concentration (EC_{50}) was defined as the concentration that reduces viral plaques by 50%, and graphically determined from the corresponding concentration-response curve.

The antiviral effect of the most effective compound por Zika was confirmed with other viral strain (ZIKA/Col, isolated from serum from a patient infected during the 2015 epidemic in Colombia[5]

by quantification of infectious viral particles in supernatants; and genome and viral protein in monolayers obtained from cells treated in a post-infection stage evaluation assay with that strain.[6]

Quantification of infectious viral particles by plaque assay

Serial dilutions of the supernatants were inoculated for 2 h on monolayers of Vero cells (1.2×10^4/well). Afterwards, the viral inoculum was removed, and 1.5% carboxymethylcellulose (Sigma-Aldrich) was added. The monolayers were fixed with 4% paraformaldehyde (Sigma-Aldrich) and stained with crystal violet after 6 days of incubation. The plaques were then counted and the results were expressed as plaque-forming units per milliliter (PFU/mL).[7]

Quantification of intracellular viral genome by qPCR

RNA was extracted from the infected and treated monolayers following the manufacturer's instructions (Zymo Quick-RNA Viral Kit). The qRT-PCR was performed from 500 ng of RNA using the qScript™ XLT One-Step RT-qPCR ToughMix®, Low ROX™ (QuantaBio) using previously described primers (1086 and 1162c with a 1107-FAB probe) that targeted a region of the envelope protein gen.[5] The samples were amplified in a QuantStudio 3 thermocycler (Thermo Scientific®) and the thermal profile used was 1 cycle at 50 °C for 2 min, 1 cycle at 95°C for 2 min, 40 cycles at 95°C for 15 s and 60°C for 1 min. For the absolute quantification of the genomic copies, we used standard curves of plasmids previously constructed.[8]

Intracellular quantification of viral protein by Cell-ELISA

The monolayers were fixed with paraformaldehyde 4% and then permeabilized with Triton X-100 (0.1%) for 30 min and then treated with 0.3% H_2O_2 prepared in 10% methanol for 30 min; nonspecific sites were then blocked with FBS (10%) for 30 min. Subsequently, the cells were

incubated overnight at 4°C with anti-NS1-ZIKV protein clone EA88 (Thermo Fisher Scientific®) mouse monoclonal antibody (Ac). The secondary antibody coupled to horseradish peroxidase (anti-mouse-HRP) was then added for 30 min, and, finally, Finally, 3,3',5,5'-tetramethylbenzidine (TMB; Invitrogen®, Carlsbad, CA, USA) was added and the absorbance was read at 620 nm using a Multiskan™ FC Microplate Photometer (Thermo Scientific®) reader.[8]

Cytotoxicity Assay. The *in vitro* cytotoxicity evaluation was performed using the 3-(4,5 dimethylthiazol2-yl)-2,5-diphenyltetrazolium bromide (MTT, Thiazolyl blue tetrazolium bromide /Sigma, Cream Ridge, NJ, USA) assay as described by Betancur-Galvis et al. (2002).[9] Briefly, Vero cells were seeded at 2.0×10^3 cells per well of 96-well plates in DMEM supplemented and incubated for 24 h at 37 ºC, 5% CO_2. Then, cells were treated with concentration of 20–160 µM of 18-(Phthalimide-2-yl) ferruginol and ribavirin for 72h or 6 days at 37 ºC, 5% CO_2. After of treatment, the media was carefully removed and 28 µL of MTT solution (4 mg/mL) was added to each well, and then it was incubated for 2 h at 37 ºC, 5% CO_2. DMSO was added to dissolve the formed formazan crystals and absorbance measure was performed in an Elisa reader (Microplate reader, BioRad), at 570 nm (OD570). The CC_{50} value (Cytotoxic Concentration 50%) were obtained by linear regression analysis of concentration-response curves.

Statistical Analysis. EC_{50} and CC_{50} values were obtained from dose effect curves for linear regression methods using the statistical GraphPad Prisma 5.0 (its values are expressed as the mean of at least four dilutions by quadruplicate). p value < 0.05 was statistically significant.

III) Molecular Docking Methodology

Molecular docking is a computational tool in which the affinity energies in a protein-ligand complex are measured.[10] This *in silico* study is used for a virtual screening of a list of molecules

with possible therapeutic effects thus facilitating the identification of active principles in proteins. In this study, free softwares were used and proteins were obtained from (https://rcsb.org) in .PDB format[11] for non-structural viral proteins from DENV-2, ZIKV, CHIKV and HHV-1 and cellular targets. The ligands (inhibitors, study molecules) are obtained from pubchem[12] (https://pubchem.ncbi.nlm.nih.gov) or drawn in Chem3D.

Three compounds ferruginol (**5**),[13] compound **6**[1,14] and methyl 14-hydroxy-dehydroabietate (**24**)[15] (Figure S1) were evaluated against cellular targets in post-infection stage with inhibitors reported in the literature, to compare results, such as Rhoa (5c4m)[16] and its inhibitor rhosin,[17] β-tubulin subunit (1JFF)[18] and its inhibitor taxol,[19] G-actin (3EKS)[20] and its inhibitor cythochalasin,[21] PI3KY (1E7U) and its inhibitor wortmannin,[22] Arp2/3 complex (3UKR) and its inhibitor CK-666,[23] Cdc42 (1KI1)[24] and its inhibitor ZCL278[25] and the last cellular target RAC1 (3TH1)[26] and its inhibitor NCS23766.[27] The above cellular targets are responsible for the translation of viral proteins and cytoskeleton rearrangement. For dengue virus the following non-structural proteins NS5 rdrp (3VWS)[28] and its inhibitor NITD-107,[29] NS5 methyltransferase (3P97) and its inhibitor SAH analogue,[30] NS3 helicase (2BMF)[31] without reported inhibitor and NS3 serine protease (3U1I)[32] and its inhibitor Bz-NKKR-H.[33] For Zika virus, the following non-structural proteins NS5 rdrp (5WZ3)[34] and its inhibitor PSI-7409,[35] NS5 methyltransferase (5M5B)[36] and its inhibitor 36A,[37] NS3 helicase (5JPS)[38] and its inhibitor GTPγ-S[39] and last NS3 serine protease (5H4I)[40] and its inhibitor Benzimidazol-1-γlmetanol[41] were selected. For chikungunya virus, the protease nsP2 (3TRK) with its inhibitor FT-0679525[42] was studied and for herpes virus type 1 (HHV-1) TK (4IVP) and its inhibitor acyclovir.[43] All binding sites depend on the chemical characteristics and are widely explained in the literature for each viral nonstructural protein and cellular target. A screening of binding pockets was performed and the corroborated ones with high binding energies

were selected. The procedure followed for the treatment of the viral protein was the identification of the active sites by means of a study of the bibliography, treatment of the viral proteins in the UCSF chimera 1.12 software[44] eliminating inactive chains, solvents and cofactors that affect the selectivity of the docking process. Later, using autodock tools 1.5.7 software, we added to the viral protein the type of atom, polar hydrogens and the gasteiger charges and finally, it was changed the format from .pdb to .pdbqt. Using the avogadro software[45] the pH is set to 7.4 and the energy is reduced to a minimum, thus guaranteeing a 3D structure suitable for molecular docking. The ligand is then treated using autodock tools 1.5.7 where polar hydrogens, rotatable bonds and the torsion point are added, and finally converted to .pdbqt format. With autodock vina 1.12 software,[46] the docking was performed and the affinity energies between the studied ligands (Fig. S1) are measured and the results are tabulated in Table S1.

Table S1. Binding energy affinities (Kcal/mol) of compounds **5**, **6** and **24** and control inhibitors for several viral and cellular targets.

Targets (PDB structure)	Binding affinity (Kcal/mol)			
Viral targets	Ferruginol **5**	Compound **6**	Compound **24**	Inhibitor
**Zika**				
NS5 rdrp (5wz3)	-7.4	-9.7	-7.1	PSI-7409 (-7.7)
NS5 methyltransferase (5m5b)	-8.1	-10.0	-7.9	36A (-8.9)
NS3 helicase (5jps)	-8.2	-9.0	-7.7	GTPγ-S (-8.6)
Complex NS2B-NS3 protease (5h4i)	-7.0	-8.7	-7.4	Benzimidazol-1-γlmethanol (-6.0)
**Dengue**				
NS5 rdrp (3vws)	-7.7	-9.6	-7.7	NITD-107 (-8.1)
NS5 methyltransferase (3p97)	-8.2	-9.5	-8.2	SAH analogue (-7.8)
NS3 helicase (2bmf)	-7.7	-9.4	-7.9	-
NS3 serine protease (3u1i)	-7.4	-8.3	-6.2	Bz-NKKR-H (-7.1)
**Chikungunya**				
Protease nsP2 (3TRK)	-7.3	-8.3	-6.6	FT-0679525 (-6.0)
**Herpes**				
HHV-1 TK (4IVP)	-7.3	-10.2	-8.1	Acyclovir (-6.2)
Cell targets				
Rhoa (5c4m)	-6.9	-9.8	-6.3	Rhosin (-9.2)
Subunit β-tubulin (1jff)	-8.4	-9.7	-8.4	Taxol (-10.0)
G-Actin (3eks)	-8.3	-9.3	-8.7	Cytochalasin D (-11.3)
PI3Kγ (1e7u)	-7.8	-8.8	-8.1	Wortmannin (-9.2)
Complex Arp2/3 (3ukr)	-8.2	-8.6	-8.0	CK-666 (-9.4)
Cdc42 (1ki1)	-7.5	-6.8	-7.1	ZCL278 (-6.5)
Rac1 (3th5)	-6.9	-6.3	-6.0	NCS23766 (-6.6)

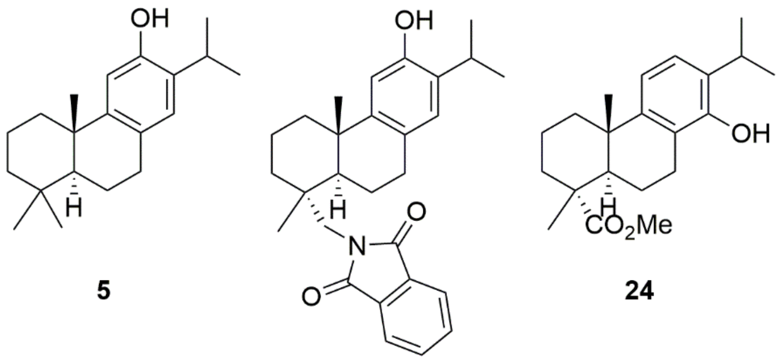

5 **6** **24**

Figure S1. Studied molecules by molecular docking.

References

1. Malkowsky, I.M.; Nieger, M.; Kataeva, O.; Waldvogel, S.R. Synthesis and properties of optically pure phenols derived from (+)-dehydroabietylamine. *Synthesis* **2007**, 773–778. https://dx.doi.org/10.1055/s-2007-965895.

2. González, M. A.; Perez-Guaita, D. Short syntheses of (+)-ferruginol from (+)-dehydroabietylamine. *Tetrahedron* **2012**, *68*, 9612–9615. https://dx.doi.org/10.1016/j.tet.2012.09.055..

3. Laiton-Donato, K.; Álvarez-Díaz, D. A.; Rengifo, A. C.; Torres-Fernández, O.; Usme-Ciro, J. A.; Rivera, J. A.; Santamaría, G.; Naizaque, J.; Monroy-Gómez, J.; Sarmiento, L.; Gunturiz, M. L.; Muñoz, A.; Vanegas, R.; Rico, A.; Pardo, L.; Peláez-Carvajal D. Complete Genome Sequence of Colombian ZikaVirus Strain Obtainedfrom BALB/c Mouse Bra in after Intraperitoneal Inoculation. *Microbiol. Resour. Announc.* **2019**, *8*, e01719-18. https://dx.doi.org/10.1128/MRA.01719-18.

4. Vlietinck, A. J.; Van Hoof, L.; Totté, J.; Lasure, A.; Vanden Berghe, D.; Rwangabo, P. C.; Mvukiyumwami, J. Screening of hundred Rwandese medicinal plants for antimicrobial and antiviral properties. *J. Ethnopharmacology* **1995**, *46*, 31–47. https://doi.org/10.1016/0378-8741(95)01226-4

5. Carrillo-Hernández, M. Y.; Ruiz-Saenz, J.; Villamizar, L. J.; Gómez-Rangel, S. Y.; Martínez-Gutierrez, M. Co-circulation and simultaneous co-infection of dengue, chikungunya, and zika viruses in patients with febrile syndrome at the Colombian-Venezuelan border. *BMC Infect. Dis.* **2018**, *18*, 61. https://doi.org/10.1186/s12879-018-2976-1.

6. Monsalve-Escudero, L. M.; Loaiza-Cano, V.; Pájaro-González, Y.; Oliveros-Díaz, A. F.; Diaz-Castillo, F.; Quiñones, W.; Robledo, S.; Martinez-Gutierrez, M. Indole alkaloids inhibit zika and chikungunya virus infection in different cell lines. *BMC Complement. Med. Ther.* **2021**, *21*, 216. https://doi.org/10.1186/s12906-021-03386-z.

7. Martinez-Gutierrez, M.; Castellanos, J. E.; Gallego-Gómez, J. C. Statins reduce dengue virus production via decreased virion assembly. *Intervirology* **2011**, *54*, 202−216. https://doi.org/10.1159/000321892.

8. Loaiza-Cano, V.; Monsalve-Escudero, L. M.; Pastrana-Restrepo, M.; Quintero-Gil, D. C.; Pulido-Muñoz, S. A.; Galeano, E.; Zapata, W.; Martinez-Gutierrez, M. In vitro and in silico anti-arboviral activities of dihalogenated phenolic derivates of L-tyrosine. *Molecules* **2021**, *26*, 3430. https://doi.org/10.3390/molecules26113430.

9. Betancur-Galvis, L.; Zuluaga, C.; Arnó, M.; González, M. A.; Zaragozá, R. J. Cytotoxic Effect (on Tumor Cells) and in Vitro Antiviral Activity against Herpes Simplex Virus of Synthetic Spongiane Diterpenes. *J. Nat. Prod.* **2002**, *65*, 189–192. https://doi.org/10.1021/np010206t

10. Schleinkofer K.; Wang T.; Wade R. C. (2006) Molecular Docking. In *Encycl. Ref. Genomics Proteomics Mol. Med.* Springer, Berlin, Heidelberg. https://doi.org/10.1007/3-540-29623-9_3820.

11. Berman, H. M.; Westbrook, J.; Feng, Z.; Gilliland, G.; Bhat, T. N.; Weissig, H.; Shindyalov, I. N.; Bourne, P. E. The Protein Data Bank. *Nucleic Acids Res.* **2000**, *28*, 235–242. https://doi.org/10.1093/nar/28.1.235.

12. Kim, S.; Chen, J.; Cheng, T.; Gindulyte, A.; He, J.; He, S.; Li, Q.; Shoemaker, B. A.; Thiessen, P. A.; Yu, B.; Zaslavsky, L.; Zhang, J.; Bolton, E. E. PubChem 2019 Update: Improved Access to Chemical Data. *Nucleic Acids Res.* **2019**, *47* (D1), D1102–D1109. https://doi.org/10.1093/nar/gky1033.

13. Saijo, H.; Kofujita, H.; Takahashi, K.; Ashitani, T. Antioxidant Activity and Mechanism of the Abietane-Type Diterpene Ferruginol. *Nat. Prod. Res.* **2015**, *29*, 1739–1743. https://doi.org/10.1080/14786419.2014.997233.

14. Roa-Linares, V. C.; Brand, Y. M.; Agudelo-Gomez, L. S.; Tangarife-Castaño, V.; Betancur-Galvis, L. A.; Gallego-Gomez, J. C.; González, M. A. Anti-Herpetic and Anti-Dengue Activity of Abietane Ferruginol Analogues Synthesized from (+)-Dehydroabietylamine. *Eur. J. Med. Chem.* **2016**, *108*, 79–88. https://doi.org/10.1016/j.ejmech.2015.11.009.

15. Zapata, B.; Rojas, M.; Betancur-Galvis, L.; Mesa-Arango, A. C.; Pérez-Guaita, D.; González, M. A. Cytotoxic, immunomodulatory, antimycotic, and antiviral activities of Semisynthetic 14-Hydroxyabietane derivatives and Triptoquinone C-4 epimers. *Med. Chem. Comm.* **2013**, *4*, 1239–1246. https://doi.org/10.1039/C3MD00151B.

16. Arthur, W. T.; Petch, L. A.; Burridge, K. Integrin Engagement Suppresses RhoA Activity via a C-Src-Dependent Mechanism. *Curr. Biol.* **2000**, *10*, 719–722. https://doi.org/10.1016/S0960-9822(00)00537-6.

17. Francis, T. C.; Gaynor, A.; Chandra, R.; Fox, M. E.; Lobo, M. K. The Selective RhoA Inhibitor Rhosin Promotes Stress Resiliency Through Enhancing D1-Medium Spiny Neuron Plasticity and Reducing Hyperexcitability. *Biol. Psychiatry* **2019**, *85*, 1001–1010. https://doi.org/10.1016/j.biopsych.2019.02.007.

18. Löwe, J.; Li, H.; Downing, K. H.; Nogales, E. Refined Structure of αβ-Tubulin at 3.5 Å Resolution. *J. Mol. Biol.* **2001**, *313*, 1045–1057. https://doi.org/10.1006/jmbi.2001.5077.

19. Gupta, M. L.; Bode, C. J.; Georg, G. I.; Himes, R. H. Understanding Tubulin - Taxol Interactions: Mutations That Impart Taxol Binding to Yeast Tubulin. *Proc. Natl. Acad. Sci. U. S. A.* **2003**, *100*, 6394–6397. https://doi.org/10.1073/pnas.1131967100.

20. Nair, U. B.; Joel, P. B.; Wan, Q.; Lowey, S.; Rould, M. A.; Trybus, K. M. Crystal Structures of Monomeric Actin Bound to Cytochalasin D. *J. Mol. Biol.* **2008**, *384*, 848–864. https://doi.org/10.1016/j.jmb.2008.09.082.

21. Brown, S. S.; Spudich, J. A. Cytochalasin Inhibits the Rate of Elongation of Actin Filament Fragments. *J. Cell Biol.* **1979**, *83* (3), 657–662. https://doi.org/10.1083/jcb.83.3.657.

22. Walker, E. H.; Pacold, M. E.; Perisic, O.; Stephens, L.; Hawkins, P. T.; Wymann, M. P.; Williams, R. L. Structural Determinants of Phosphoinositide 3-Kinase Inhibition by Wortmannin, LY294002, Quercetin, Myricetin, and Staurosporine. *Mol. Cell* **2000**, *6*, 909–919. https://doi.org/10.1016/S1097-2765(05)00089-4.

23. Baggett, A. W.; Cournia, Z.; Han, M. S.; Patargias, G.; Glass, A. C.; Liu, S. Y.; Nolen, B. J. Structural Characterization and Computer-Aided Optimization of a Small-Molecule Inhibitor of the Arp2/3 Complex, a Key Regulator of the Actin Cytoskeleton. *ChemMedChem* **2012**, *7*, 1286–1294. https://doi.org/10.1002/cmdc.201200104.

24. Snyder, J. T.; Worthylakel, D. K.; Rossman, K. L.; Betts, L.; Pruitt, W. M.; Siderovski, D. P.; Der, C. J.; Sondek, J. Structural Basis for the Selective Activation of Rho Gtpases by Dbl Exchange Factors. *Nature Struct. Biol.* **2002**, *9*, 468–475. https://doi.org/10.1038/nsb796.

25. Muhoza, D.; Adams, P. D. Two Small Molecules, ZCL278 and AZA197 Show Promise in Influencing Protein Interactions Involving the Ras-Related Protein Cell Division Cycle 42 [Cdc42] to Modulate Its Oncogenic Potential. *Open J. Biophys.* **2017**, *7*, 71–81. https://doi.org/10.4236/ojbiphy.2017.73006.

26. Krauthammer, M.; Kong, Y.; Ha, B. H.; Evans, P.; Bacchiocchi, A.; McCusker, J. P.; Cheng, E.; Davis, M. J.; Goh, G.; Choi, M.; Ariyan, S.; Narayan, D.; Dutton-Regester, K.; Capatana, A.; Holman, E. C.; Bosenberg, M.; Sznol, M.; Kluger, H. M.; Brash, D. E.; Stern, D. F.; Materin, M. A.; Lo, R. S.; Mane, S.; Ma, S.; Kidd, K. K.; Hayward, N. K.; Lifton, R. P.; Schlessinger, J.; Boggon, T. J.; Halaban, R. Exome Sequencing Identifies Recurrent Somatic RAC1 Mutations in Melanoma. *Nature Genet.* **2012**, *44*, 1006–1014. https://doi.org/10.1038/ng.2359.

27. Connolly, M.; Veale, D. J.; Fearon, U. Acute Serum Amyloid A Regulates Cytoskeletal Rearrangement, Cell Matrix Interactions and Promotes Cell Migration in Rheumatoid Arthritis. *Ann. Rheum. Dis.* **2011**, *70*, 1296–1303. https://doi.org/10.1136/ard.2010.142240.

28. Noble, C. G.; Lim, S. P.; Chen, Y.-L.; Liew, C. W.; Yap, L.; Lescar, J.; Shi, P.-Y. Conformational Flexibility of the Dengue Virus RNA-Dependent RNA Polymerase Revealed by a Complex with an Inhibitor. *J. Virol.* **2013**, *87*, 5291–5295. https://doi.org/10.1128/jvi.00045-13.

29. Anusuya, S.; Velmurugan, D.; Gromiha, M. M. Identification of Dengue Viral RNA-Dependent RNA Polymerase Inhibitor Using Computational Fragment-Based Approaches and Molecular Dynamics Study. *J. Biomol. Struct. Dynam.* **2016**, *34*, 1512–1532. https://doi.org/10.1080/07391102.2015.1081620.

30. Lim, S. P.; Sonntag, L. S.; Noble, C.; Nilar, S. H.; Ng, R. H.; Zou, G.; Monaghan, P.; Chung, K. Y.; Dong, H.; Liu, B.; Bodenreider, C.; Lee, G.; Ding, M.; Chan, W. L.; Wang, G.; Jian, Y. L.; Chao, A. T.; Lescar, J.; Yin, Z.; Vedananda, T. R.; Keller, T. H.; Shi, P.-Y. Small Molecule Inhibitors That Selectively Block Dengue Virus Methyltransferase. *J. Biol. Chem.* **2011**, *286*, 6233–6240. https://doi.org/10.1074/jbc.M110.179184.

31. Xu, T.; Sampath, A.; Chao, A.; Wen, D.; Nanao, M.; Chene, P.; Vasudevan, S. G.; Lescar, J. Structure of the Dengue Virus Helicase/Nucleoside Triphosphatase Catalytic Domain at a Resolution of 2.4 Å. *J. Virol.* **2005**, *79*, 10278–10288. https://doi.org/10.1128/jvi.79.16.10278-10288.2005.

32. Noble, C. G.; Seh, C. C.; Chao, A. T.; Shi, P. Y. Ligand-Bound Structures of the Dengue Virus Protease Reveal the Active Conformation. *J. Virol.* **2012**, *86*, 438–446. https://doi.org/10.1128/jvi.06225-11.

33. Schüller, A.; Yin, Z.; Brian Chia, C. S.; Doan, D. N. P.; Kim, H.-K.; Shang, L.; Loh, T. P.; Hill, J.; Vasudevan, S. G. Tripeptide Inhibitors of Dengue and West Nile Virus NS2B-NS3 Protease. *Antiviral Res.* **2011**, *92*, 96–101. https://doi.org/10.1016/j.antiviral.2011.07.002.

34. Duan, W.; Song, H.; Wang, H.; Chai, Y.; Su, C.; Qi, J.; Shi, Y.; Gao, G. F. The Crystal Structure of Zika Virus NS 5 Reveals Conserved Drug Targets. *EMBO J.* **2017**, *36*, 919–933. https://doi.org/10.15252/embj.201696241.

35. Lin, Y.; Zhang, H.; Song, W.; Si, S.; Han, Y.; Jiang, J. Identification and Characterization of Zika Virus NS5 RNA-Dependent RNA Polymerase Inhibitors. *Int. J. Antimicrob. Agents* **2019**, *54* (4), 502–506. https://doi.org/10.1016/j.ijantimicag.2019.07.010.

36. Coutard, B.; Barral, K.; Lichière, J.; Selisko, B.; Martin, B.; Aouadi, W.; Lombardia, M. O.; Debart, F.; Vasseur, J.-J.; Guillemot, J. C.; Canard, B.; Decroly, E. Zika Virus Methyltransferase: Structure and Functions for Drug Design Perspectives. *J. Virol.* **2017**, *91*, e02202-16. https://doi.org/10.1128/jvi.02202-16.

37. Liu, X.; Zhao, Y.; Zhang, J. Z. H. Molecular Mechanism of Ligand Bindings to Zika Virus at SAM Site. *Chem. Phys. Lett.* **2019**, *735*, 136771. https://doi.org/10.1016/j.cplett.2019.136771.

38. Shankar, A.; Patil, A. A.; Skariyachan, S. Recent Perspectives on Genome, Transmission, Clinical Manifestation, Diagnosis, Therapeutic Strategies, Vaccine Developments, and Challenges of Zika Virus Research. *Front. Microbiol.* **2017**, *8*, 1761. https://doi.org/10.3389/fmicb.2017.01761.

39. Cao, X.; Li, Y.; Jin, X.; Li, Y.; Guo, F.; Jin, T. Molecular Mechanism of Divalent-Metal-Induced Activation of NS3 Helicase and Insights into Zika Virus Inhibitor Design. *Nucleic Acids Res.* **2016**, *44*, 10505–10514. https://doi.org/10.1093/nar/gkw941.

40. Zhang, Z.; Li, Y.; Loh, Y. R.; Phoo, W. W.; Hung, A. W.; Kang, C. B.; Luo, D. Crystal structure of unlinked NS2B-NS3 Protease from Zika Virus. *Science* **2016**, *354*, 1597–1600. https://doi.org/10.1126/science.aai9309.

41. Coronado, M. A.; Eberle, R. J.; Bleffert, N.; Feuerstein, S.; Olivier, D. S.; de Moraes, F. R.; Willbold, D.; Arni, R. K. Zika Virus NS2B/NS3 Proteinase: A New Target for an Old Drug - Suramin a Lead Compound for NS2B/NS3 Proteinase Inhibition-. *Antiviral Res.* **2018**, *160*, 118–125. https://doi.org/10.1016/j.antiviral.2018.10.019.

42. Jadav, S. S.; Sinha, B. N.; Hilgenfeld, R.; Pastorino, B.; De Lamballerie, X.; Jayaprakash, V. Thiazolidone Derivatives as Inhibitors of Chikungunya Virus. *Eur. J. Med. Chem.* **2015**, *89*, 172–178. https://doi.org/10.1016/j.ejmech.2014.10.042.

43. Tenser, R. B. Role of Herpes Simplex Virus Thymidine Kinase Expression In Viral Pathogenesis and Latency. *Intervirology* **1991**, *32*, 76–92. https://doi.org/10.1159/000150188.

44. Pettersen, E. F.; Goddard, T. D.; Huang, C. C.; Couch, G. S.; Greenblatt, D. M.; Meng, E. C.; Ferrin, T. E. UCSF Chimera - A Visualization System for Exploratory Research and Analysis. *J. Comput. Chem.* **2004**, *25*, 1605–1612. https://doi.org/10.1002/jcc.20084.

45. Hanwell, M. D.; Curtis, D. E.; Lonie, D. C.; Vandermeersch, T.; Zurek, E.; Hutchison, G. R. Avogadro: An Advanced Semantic Chemical Editor, Visualization, and Analysis Platform. *J. Cheminform.* **2012**, *4*, 17. https://doi.org/10.1186/1758-2946-4-17.

46. Trott, O.; Olson, A. J. Autodock Vina: Improving the Speed and Accuracy of Docking with a New Scoring Function, Efficient Optimization and Multithreading. *J. Comput. Chem.* **2010**, *30*, 455–461. https://doi.org/10.1002/jcc.21334.

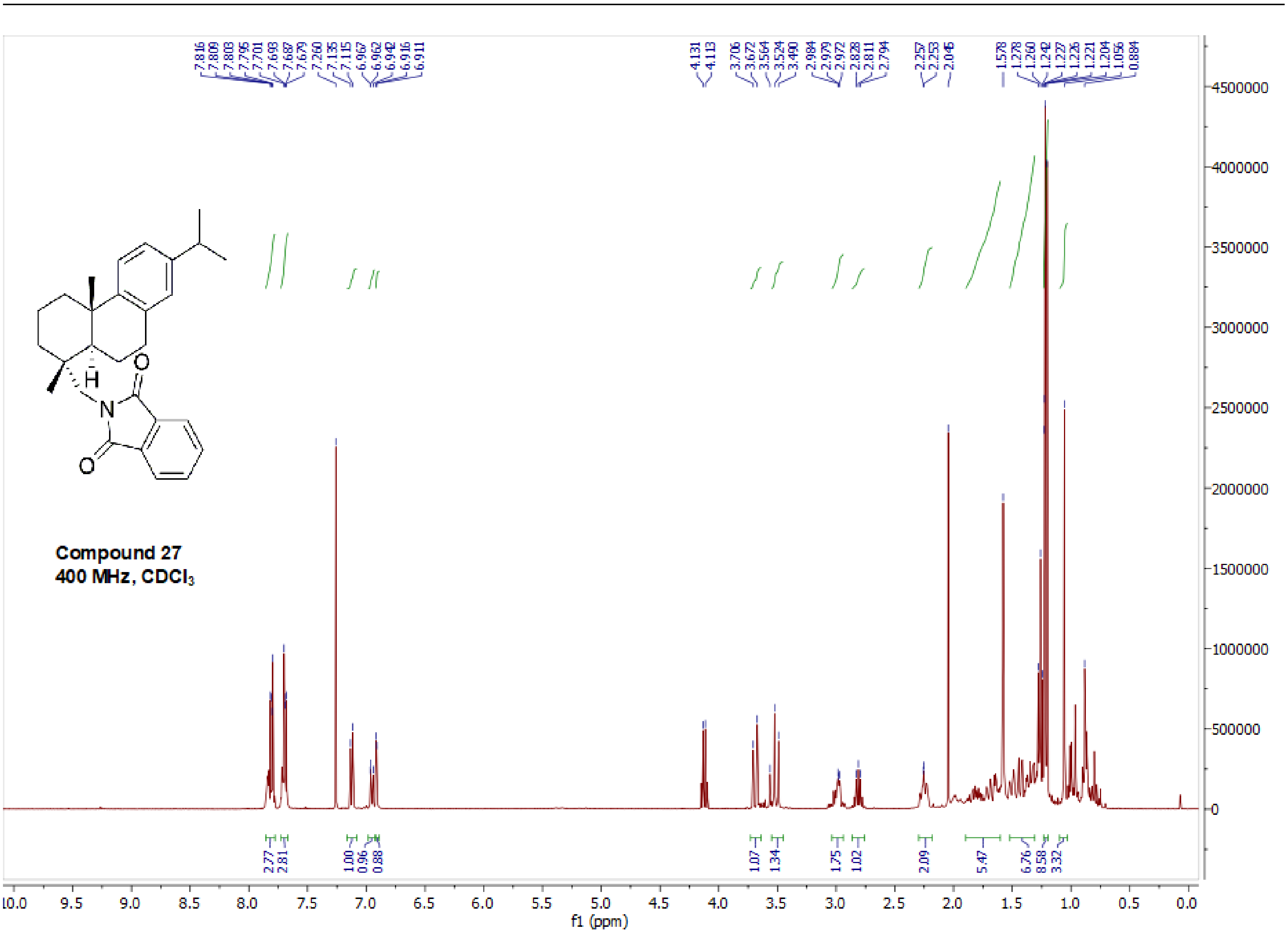
Compound 27
400 MHz, CDCl3
f1 (ppm)

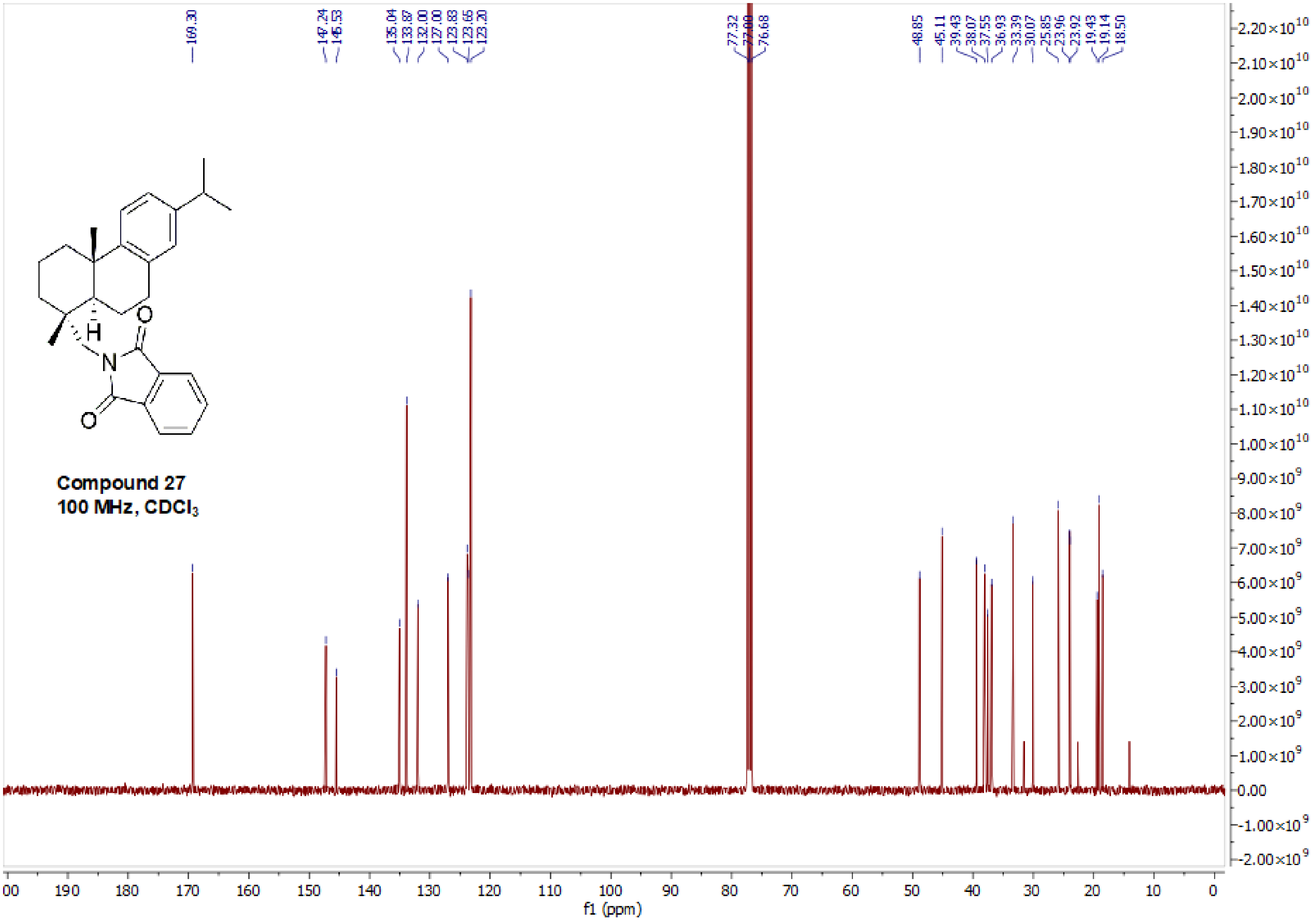

Compound 27
100 MHz, CDCl3
169.30
147.24
145.53
135.04
133.87
132.00
127.00
123.83
123.65
123.20
77.32
77.00
76.68
48.85
45.11
39.43
38.07
37.55
36.93
33.39
30.07
25.85
23.96
23.92
19.43
19.14
18.50
f1 (ppm)

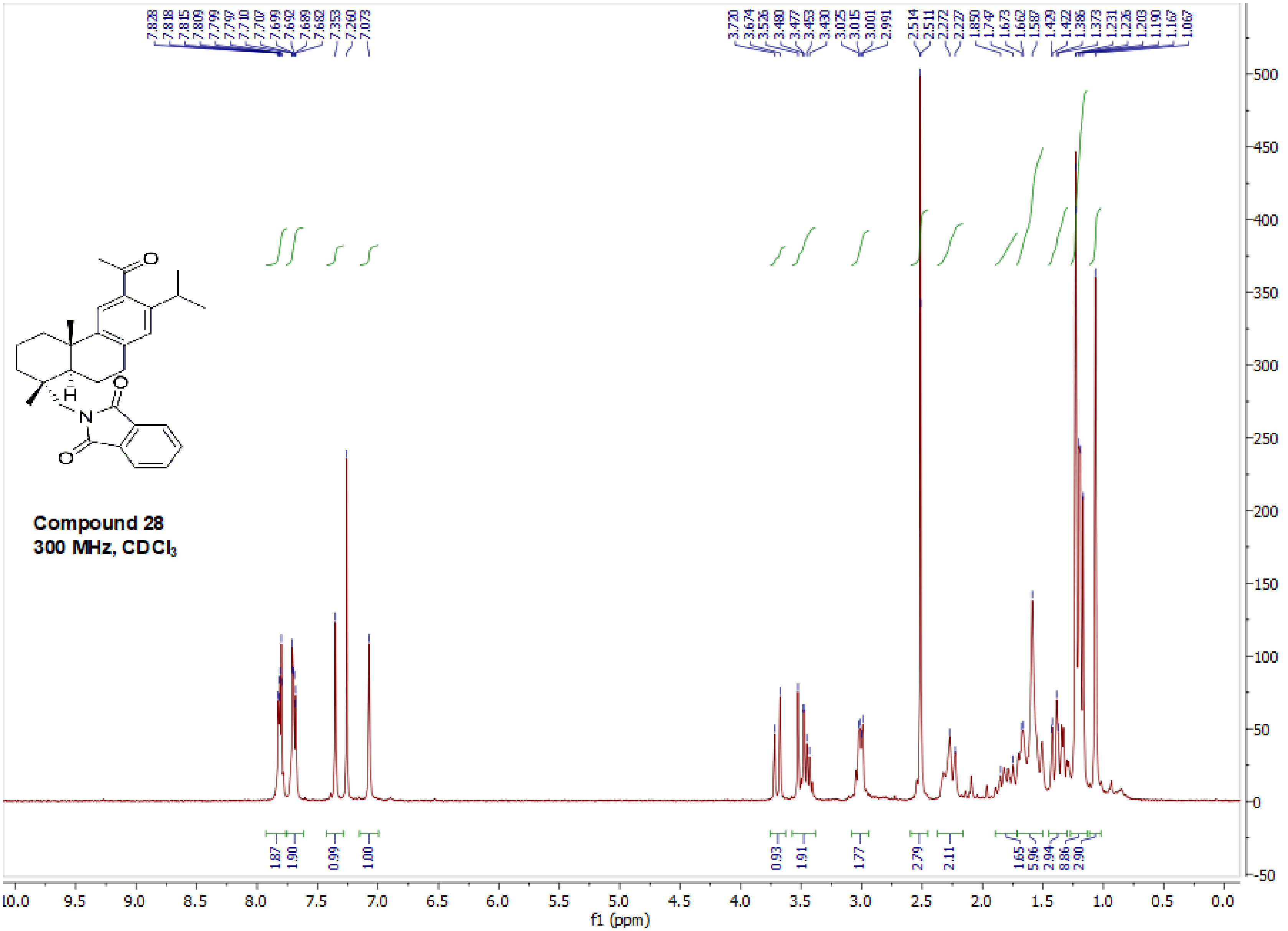
Compound 28
300 MHz, CDCl3
f1 (ppm)

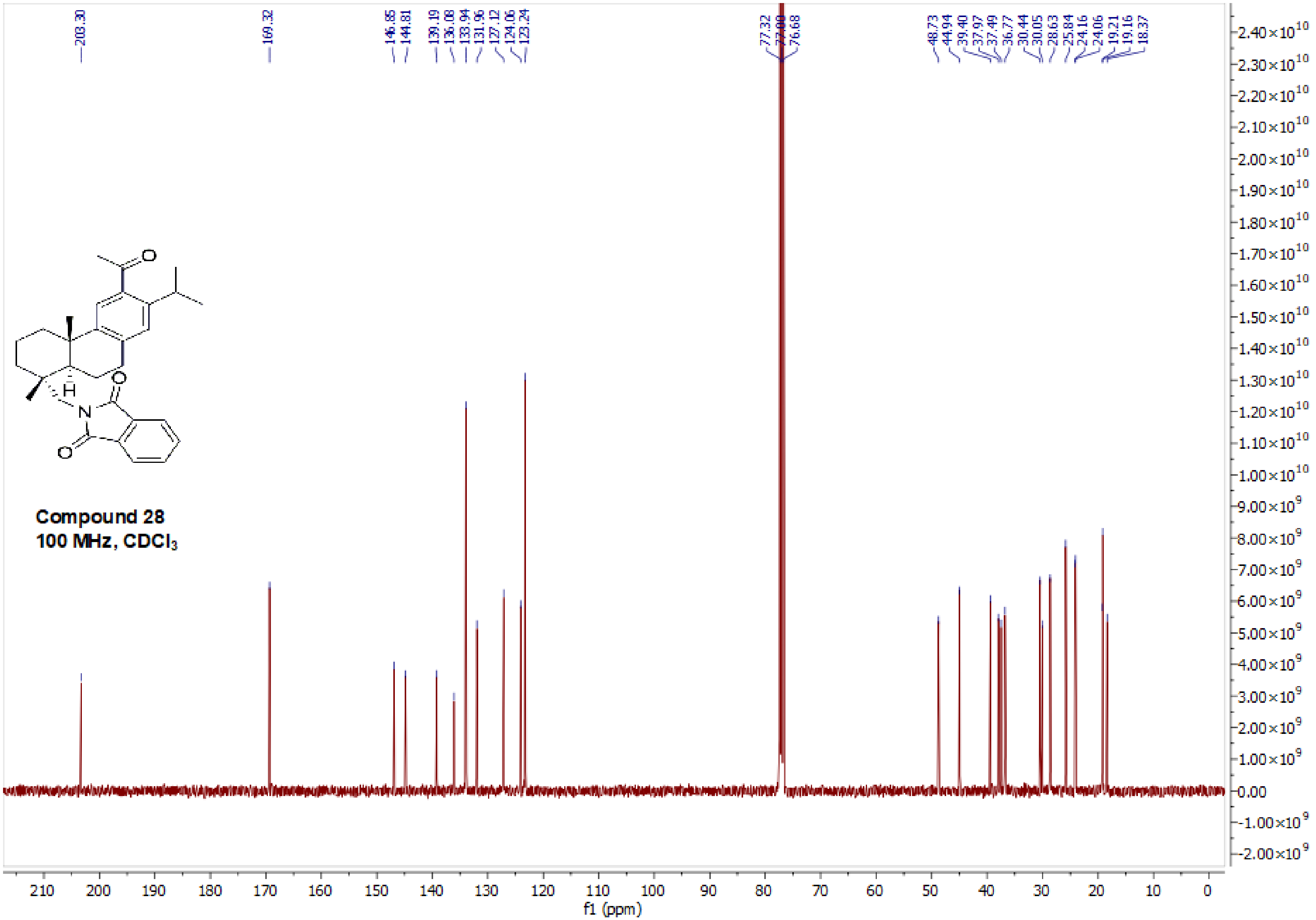
203.30
169.32
146.85
144.81
139.19
136.08
133.94
131.96
127.12
124.06
123.24
77.32
77.00
76.68
48.73
44.94
39.40
37.97
37.49
36.77
30.44
30.05
28.63
25.84
24.16
24.06
19.21
19.16
18.37
Compound 28
100 MHz, CDCl3
f1 (ppm)

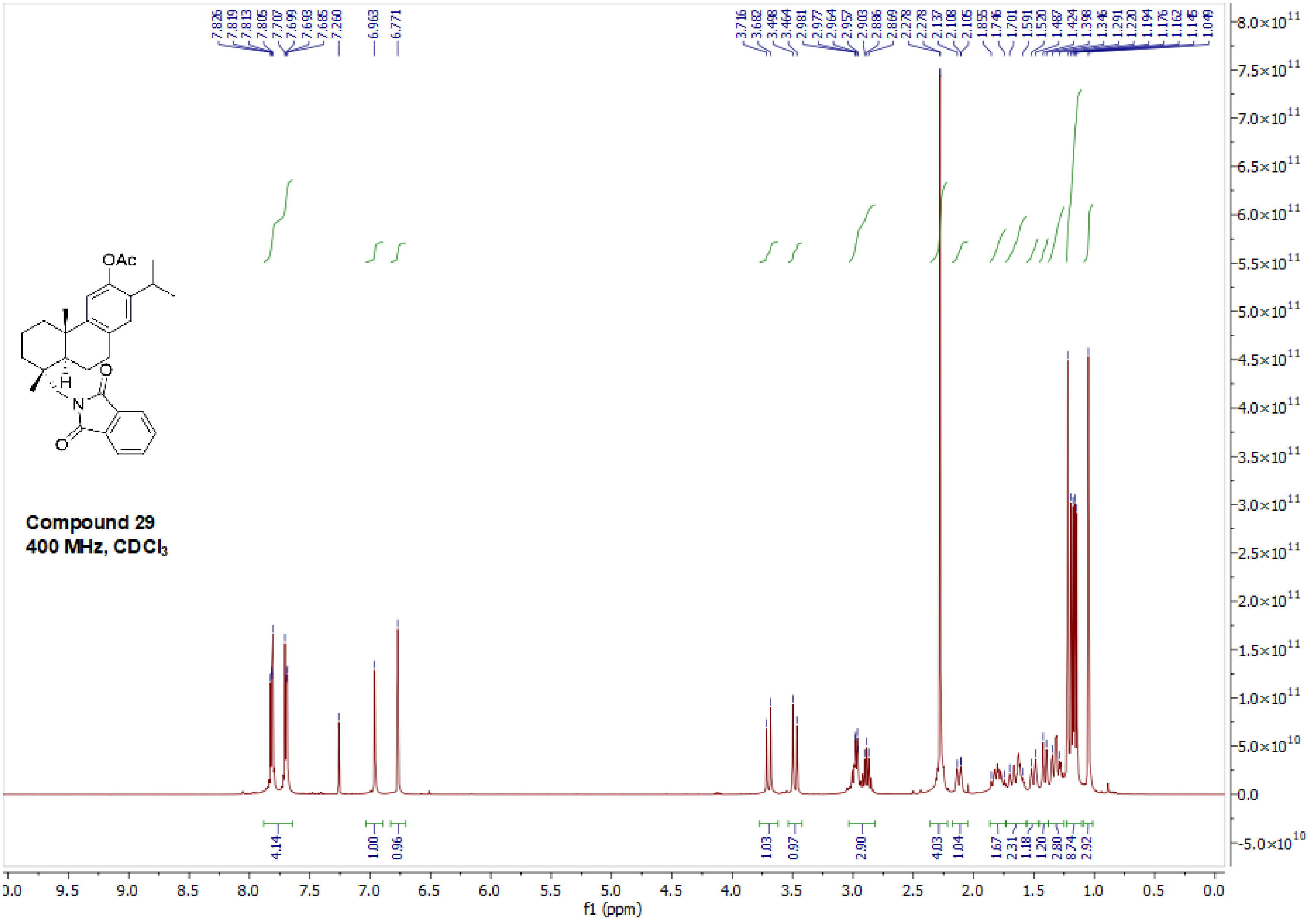
7.826
7.819
7.813
7.805
7.707
7.699
7.693
7.685
7.260
6.963
6.771
3.716
3.682
3.498
3.464
2.981
2.977
2.964
2.957
2.903
2.886
2.869
2.278
2.278
2.137
2.108
2.105
1.855
1.746
1.701
1.591
1.520
1.487
1.424
1.398
1.346
1.291
1.220
1.194
1.176
1.162
1.145
1.049
OAc
H
N
O
O
Compound 29
400 MHz, CDCl3
4.14
1.00
0.96
1.03
0.97
2.90
4.03
1.04
1.67
2.31
1.18
1.20
2.80
8.74
2.92
f1 (ppm)

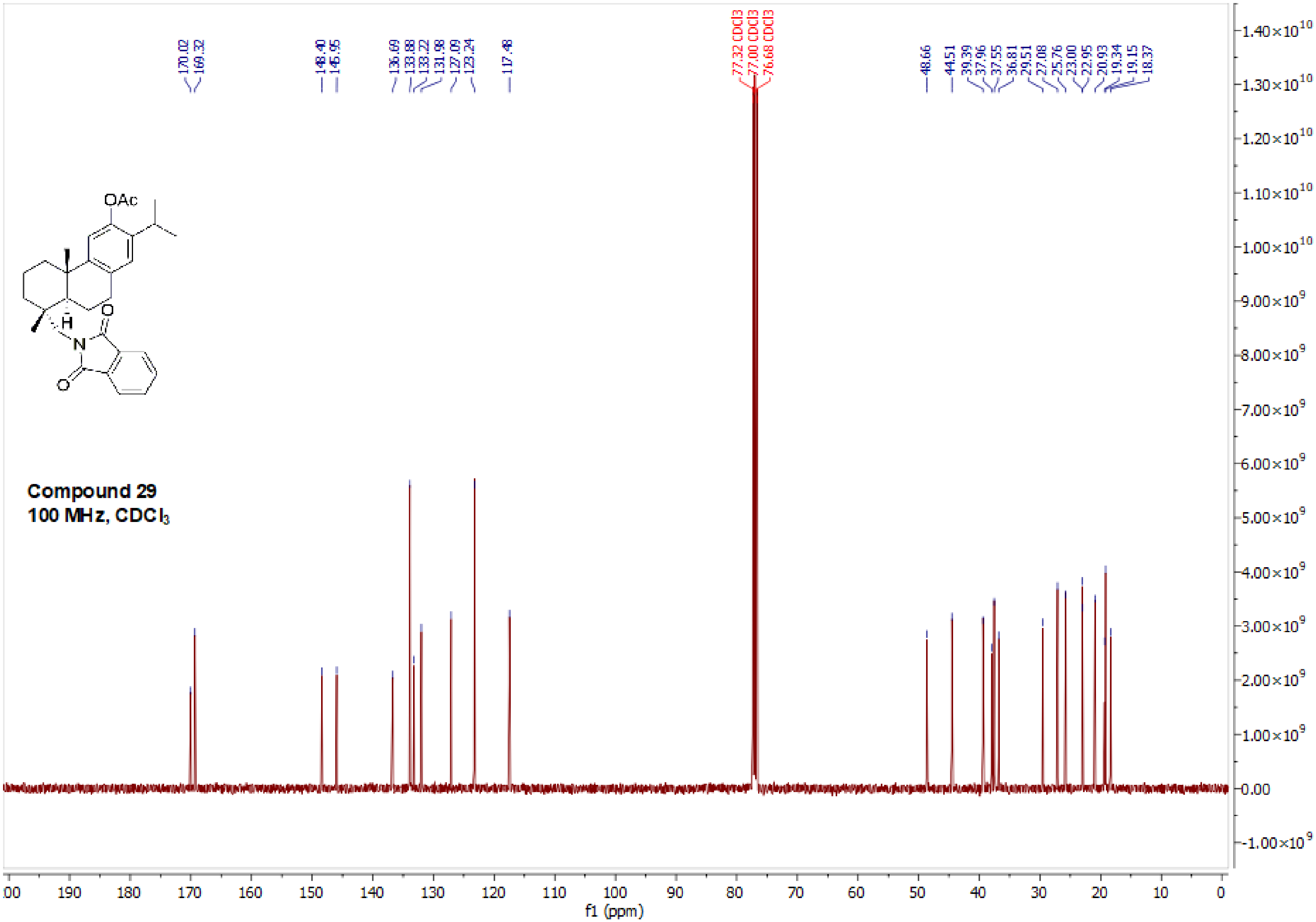
170.02
169.32
148.40
145.95
136.69
133.88
133.22
131.98
127.09
123.24
117.48
77.32 CDCl3
77.00 CDCl3
76.68 CDCl3
48.66
44.51
39.39
37.96
37.55
36.81
29.51
27.08
25.76
23.00
22.95
20.93
19.34
19.15
18.37
OAc
H
N
O
O
Compound 29
100 MHz, CDCl3
f1 (ppm)

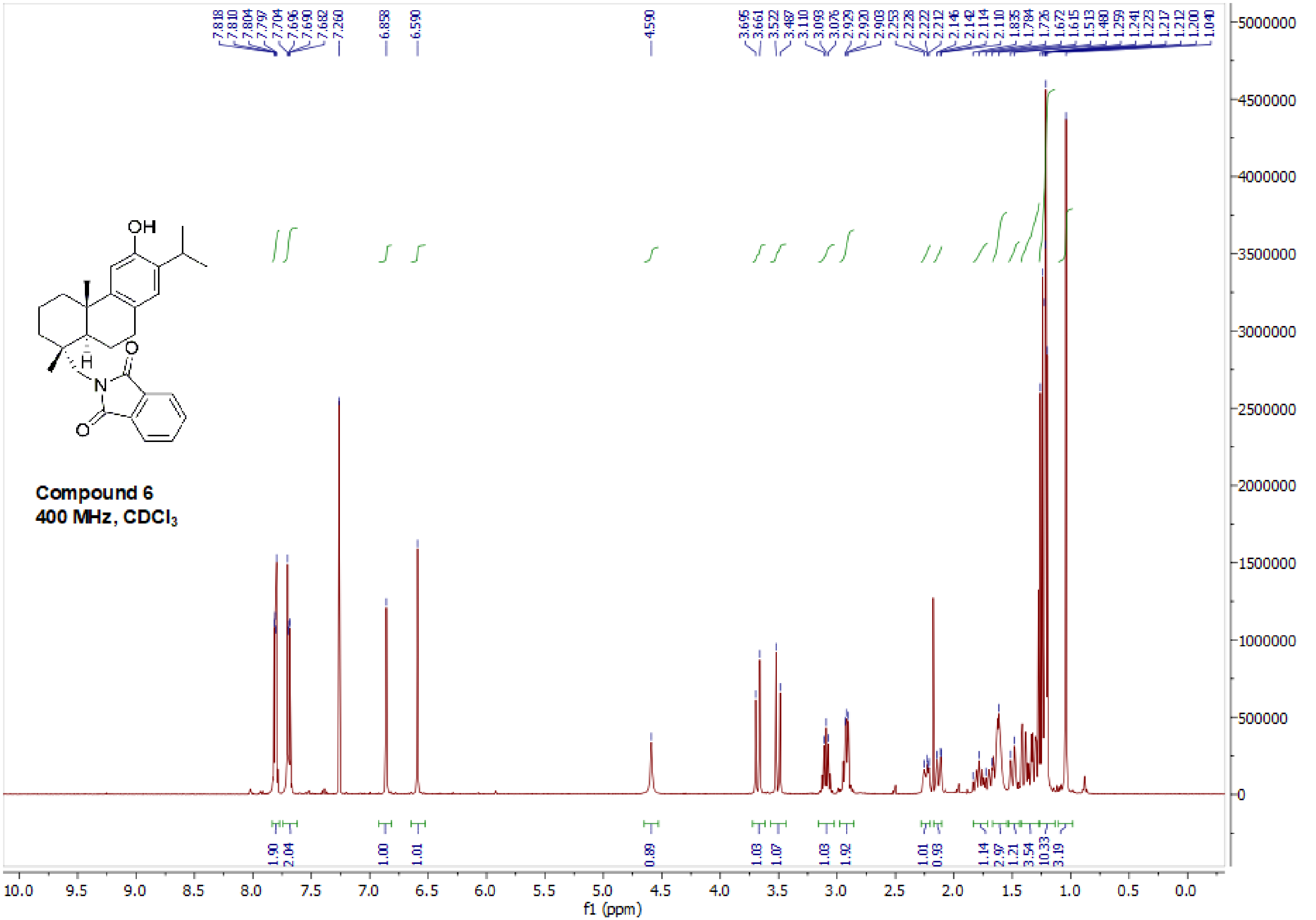
Compound 6
400 MHz, CDCl3
f1 (ppm)

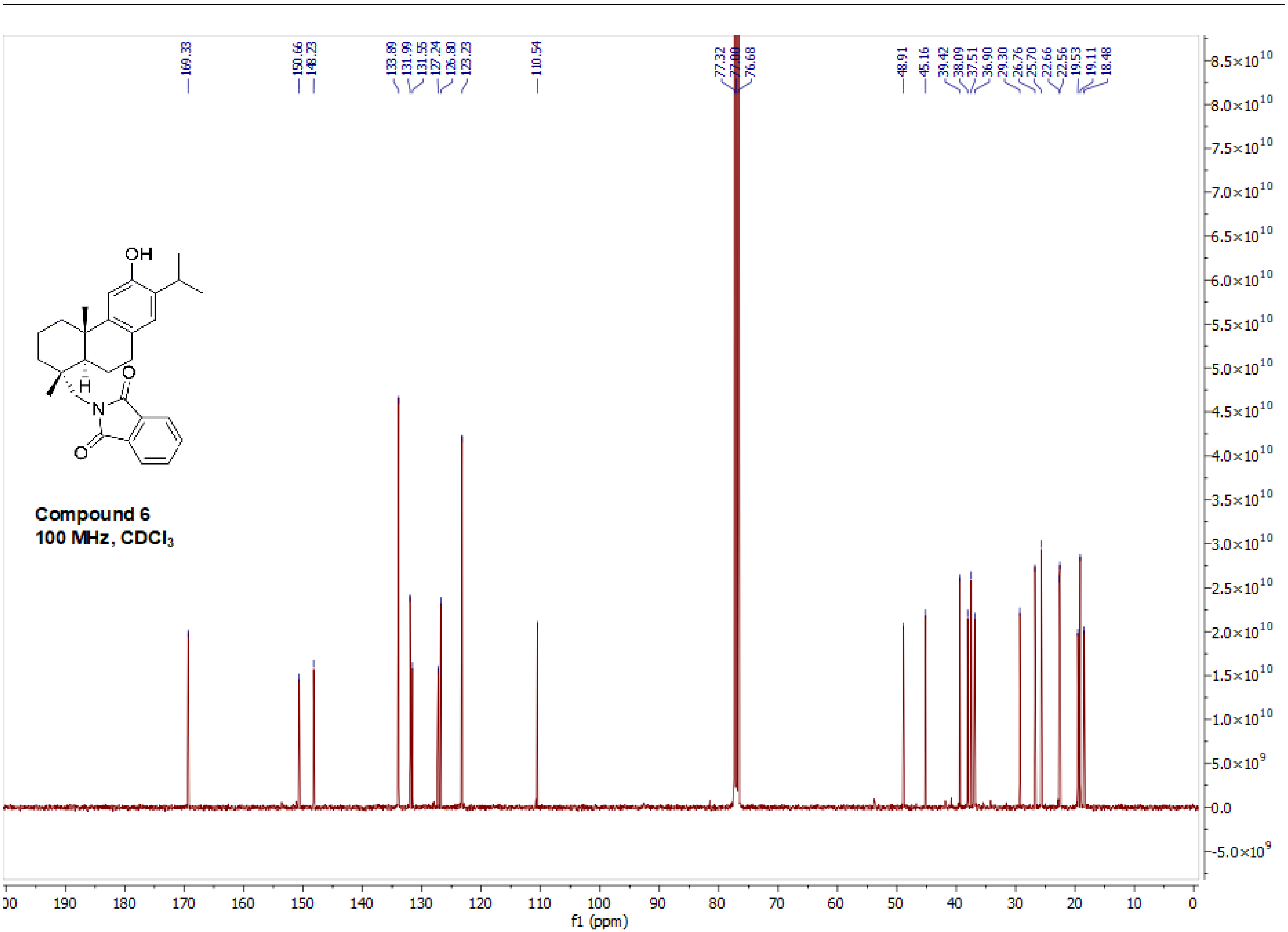
169.33
150.66
148.23
133.89
131.99
131.55
127.24
126.80
123.23
110.54
77.32
77.00
76.68
48.91
45.16
39.42
38.09
37.51
36.90
29.30
26.76
25.70
22.66
22.56
19.53
19.11
18.48
OH
H
N
O
O
Compound 6
100 MHz, CDCl3
f1 (ppm)

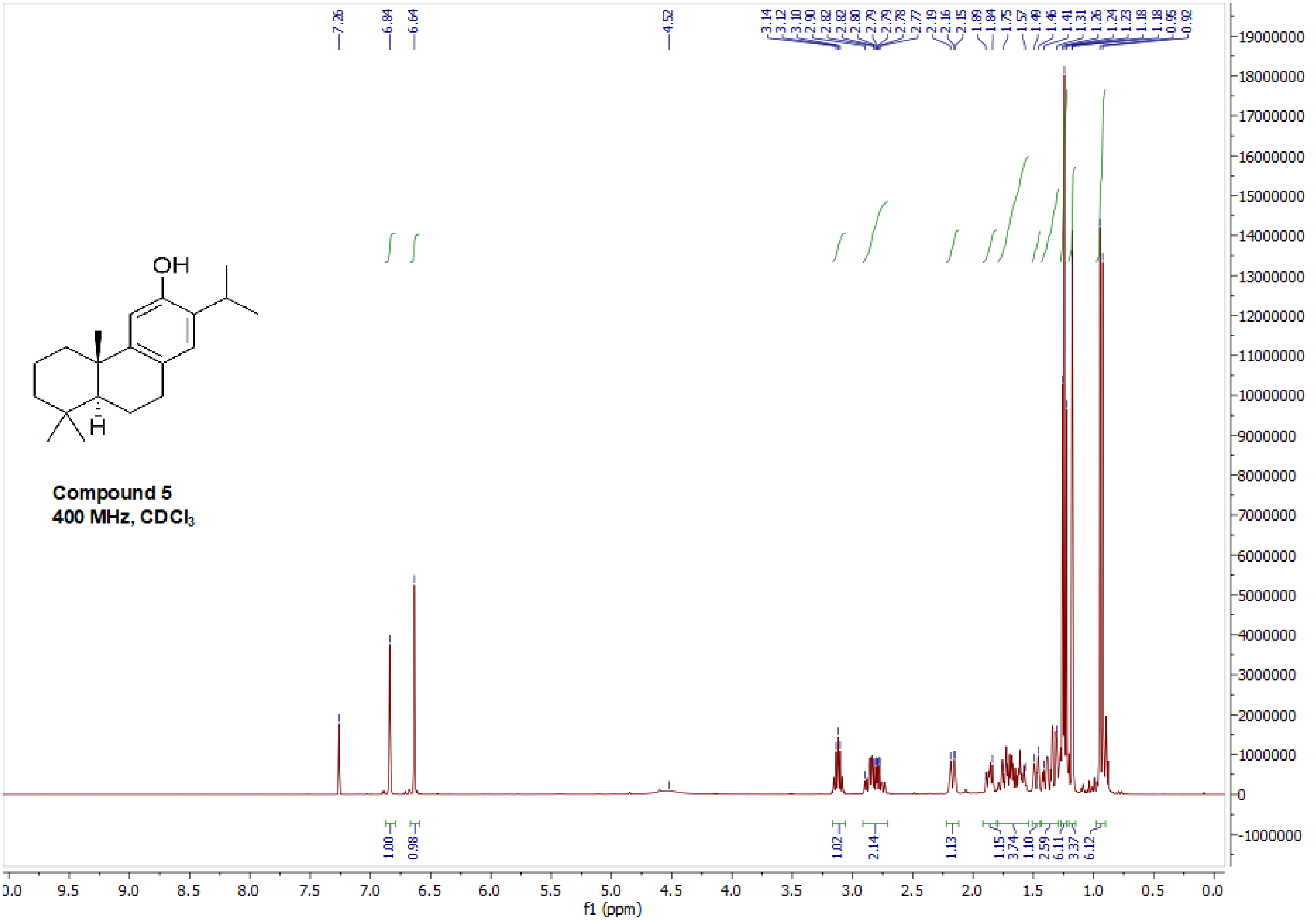

OH
H
Compound 5
400 MHz, CDCl3
f1 (ppm)

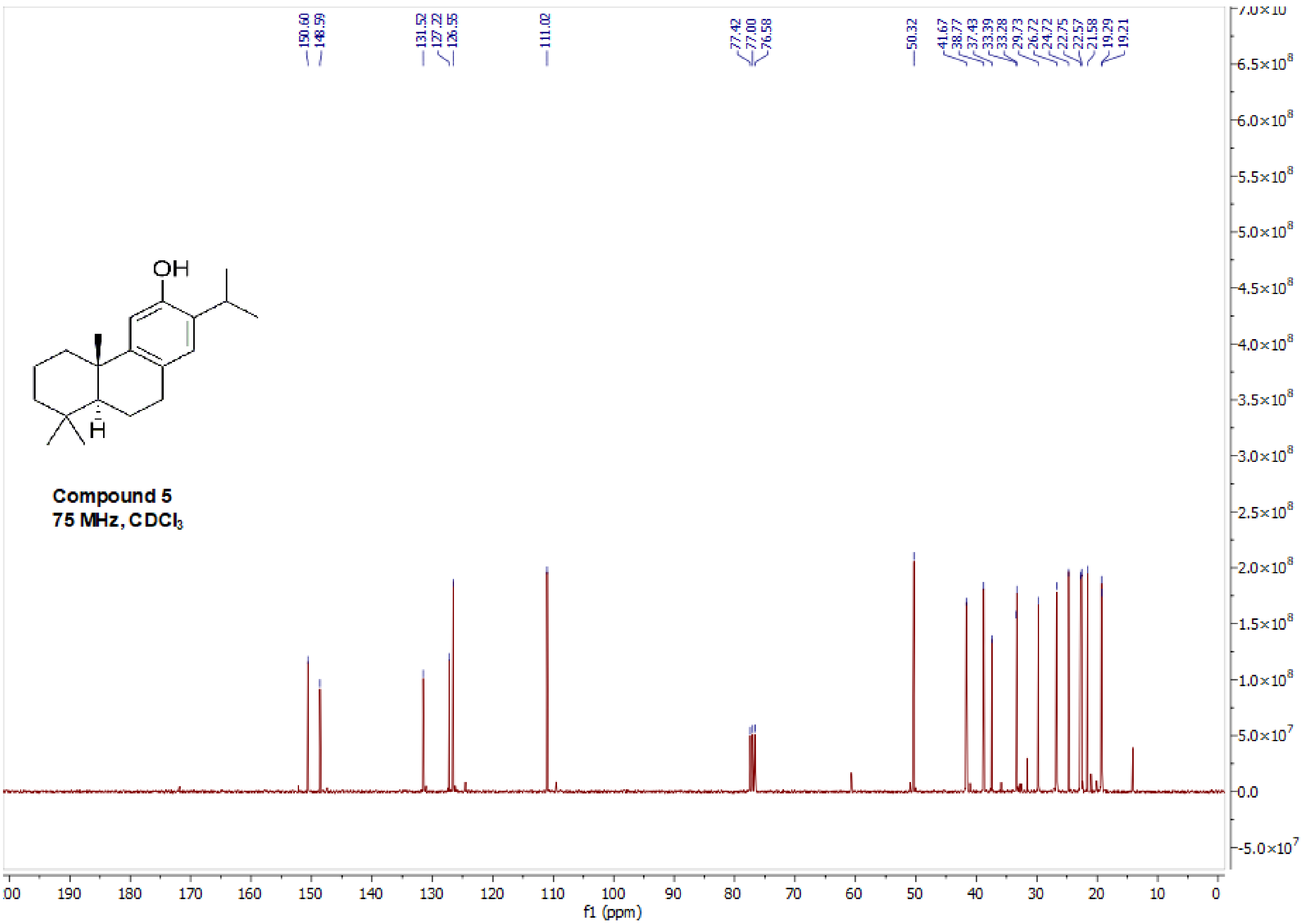
150.60
148.59
131.52
127.22
126.55
111.02
77.42
77.00
76.58
50.32
41.67
38.77
37.43
33.39
33.28
29.73
26.72
24.72
22.75
22.57
21.58
19.29
19.21
OH
H
Compound 5
75 MHz, CDCl3
f1 (ppm)

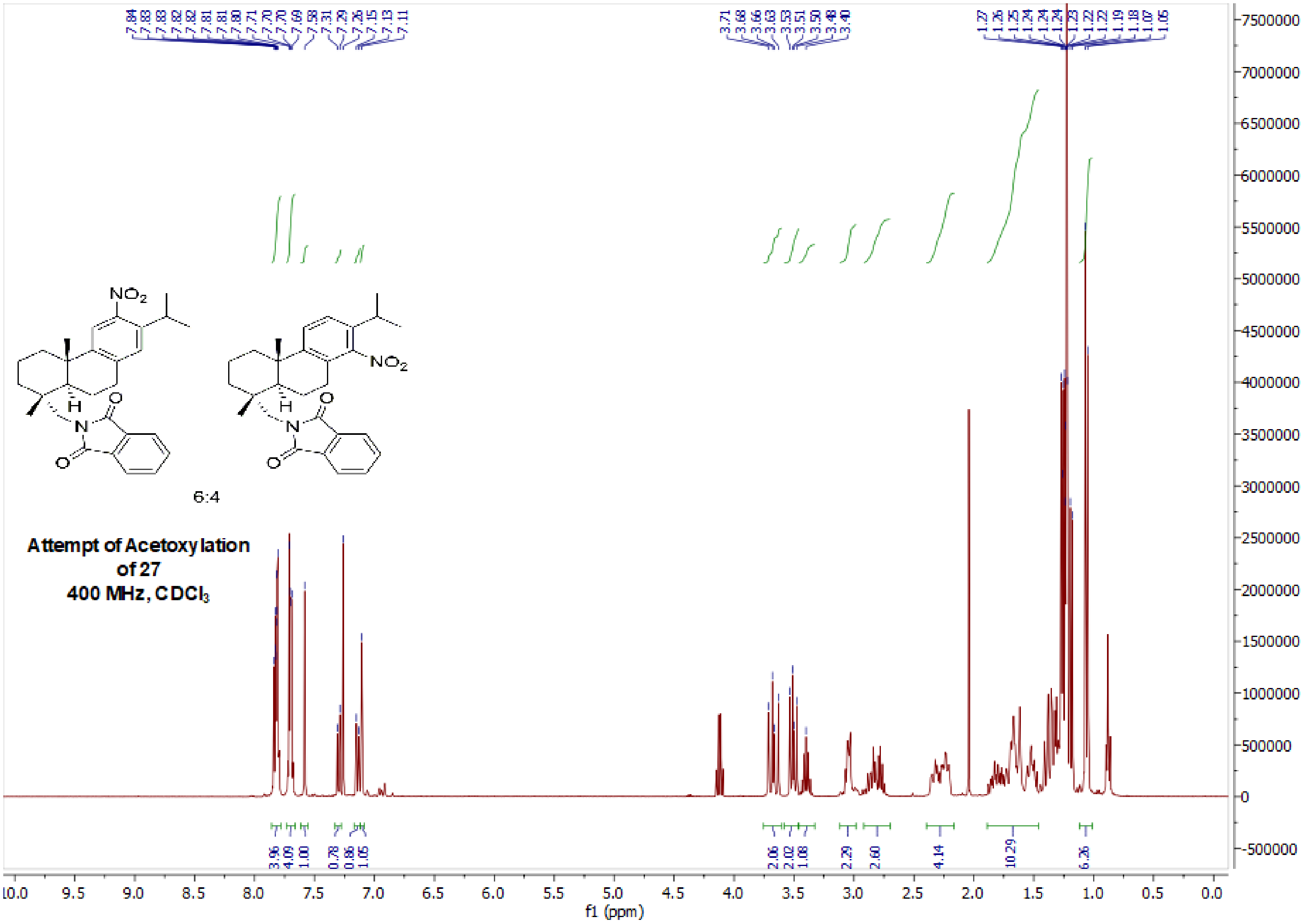

^{1}H NMR spectrum (400MHz, $CDCl_3$) of the products in the attempt of direct acetoxylation of **27**.
